# Immunomodulatory role of reactive oxygen species and nitrogen species during T cell-driven neutrophil-enriched acute and chronic cutaneous delayed-type hypersensitivity reactions

**DOI:** 10.7150/thno.51462

**Published:** 2021-01-01

**Authors:** Roman Mehling, Johannes Schwenck, Christina Lemberg, Christoph Trautwein, Laimdota Zizmare, Daniela Kramer, Anne Müller, Birgit Fehrenbacher, Irene Gonzalez-Menendez, Leticia Quintanilla-Martinez, Katrin Schröder, Ralph P. Brandes, Martin Schaller, Wolfram Ruf, Martin Eichner, Kamran Ghoreschi, Martin Röcken, Bernd J. Pichler, Manfred Kneilling

**Affiliations:** 1 Werner Siemens Imaging Center, Department of Preclinical Imaging and Radiopharmacy, Eberhard Karls University, Tübingen, Germany.; 2 Department of Nuclear Medicine and Clinical Molecular Imaging, Eberhard Karls University, Tübingen, Germany.; 3 Cluster of Excellence iFIT (EXC 2180) "Image-Guided and Functionally Instructed Tumor Therapies", Eberhard Karls University, 72076 Tübingen, Germany; 4 Section of Immunology, Vetsuisse Faculty, University of Zürich, Zürich, Switzerland.; 5 Interfaculty Institute for Biochemistry, Eberhard Karls University, Tübingen, Germany.; 6 Department of Dermatology, Eberhard Karls University, Tübingen, Germany.; 7 Department of Pathology, Eberhard Karls University, Tübingen, Germany; 8 Institute for Cardiovascular Physiology, Goethe-University, Frankfurt, Germany.; 9 Center for Thrombosis and Hemostasis, University Medical Center Mainz, Mainz, Germany.; 10 Institute for Clinical Epidemiology and Applied Biometry, Eberhard Karls University, Tübingen, Germany; 11 Department of Dermatology, Venereology and Allergology, Charité - Universitätsmedizin Berlin, Berlin, Germany.

**Keywords:** DTHR, Neutrophils, ROS/RNS, NETs, acute chronic inflammation

## Abstract

**Rationale:** Reactive oxygen species (ROS) and reactive nitrogen species (RNS) are important regulators of inflammation. The exact impact of ROS/RNS on cutaneous delayed-type hypersensitivity reaction (DTHR) is controversial. The aim of our study was to identify the dominant sources of ROS/RNS during acute and chronic trinitrochlorobenzene (TNCB)-induced cutaneous DTHR in mice with differently impaired ROS/RNS production.

**Methods:** TNCB-sensitized wild-type, NADPH oxidase 2 (NOX2)- deficient (gp91^phox-/-^), myeloperoxidase-deficient (MPO^-/-^), and inducible nitric oxide synthase-deficient (iNOS^-/-^) mice were challenged with TNCB on the right ear once to elicit acute DTHR and repetitively up to five times to induce chronic DTHR. We measured ear swelling responses and noninvasively assessed ROS/RNS production *in vivo* by employing the chemiluminescence optical imaging (OI) probe L-012. Additionally, we conducted extensive *ex vivo* analyses of inflamed ears focusing on ROS/RNS production and the biochemical and morphological consequences.

**Results:** The *in vivo* L-012 OI of acute and chronic DTHR revealed completely abrogated ROS/RNS production in the ears of gp91^phox-/-^ mice, up to 90 % decreased ROS/RNS production in the ears of MPO^-/-^ mice and unaffected ROS/RNS production in the ears of iNOS^-/-^ mice. The DHR flow cytometry analysis of leukocytes derived from the ears with acute DTHR confirmed our *in vivo* L-012 OI results. Nevertheless, we observed no significant differences in the ear swelling responses among all the experimental groups. The histopathological analysis of the ears of gp91^phox-/-^ mice with acute DTHRs revealed slightly enhanced inflammation. In contrast, we observed a moderately reduced inflammatory immune response in the ears of gp91^phox-/-^ mice with chronic DTHR, while the inflamed ears of MPO^-/-^ mice exhibited the strongest inflammation. Analyses of lipid peroxidation, 8-hydroxy-2'deoxyguanosine levels, redox related metabolites and genomic expression of antioxidant proteins revealed similar oxidative stress in all experimental groups. Furthermore, inflamed ears of wild-type and gp91^phox-/-^ mice displayed neutrophil extracellular trap (NET) formation exclusively in acute but not chronic DTHR.

**Conclusions:** MPO and NOX2 are the dominant sources of ROS/RNS in acute and chronic DTHR. Nevertheless, depletion of one primary source of ROS/RNS exhibited only marginal but conflicting impact on acute and chronic cutaneous DTHR. Thus, ROS/RNS are not a single entity, and each species has different properties at certain stages of the disease, resulting in different outcomes.

## Introduction

Polymorphonuclear neutrophils (PMNs) are the most abundant circulating leukocytes and are the first immune cells to home to sites of inflammation after an inflammatory stimulus. PMNs are crucial regulators of the immune response and are essential for killing invading pathogens. Moreover, PMNs are heavily involved in the pathology of several T cell-mediated autoimmune diseases, such as rheumatoid arthritis, psoriasis vulgaris, multiple sclerosis and systemic lupus erythematosus 1. One of the mechanisms by which PMNs affect innate and adaptive immunity is the generation of ROS and RNS, leading to an oxidative burst 2-4. NADPH oxidase (NOX2) is the primary source of ROS in PMNs and produces superoxide anions by the one-electron reduction of dioxygen 5, 6. Superoxide (O_2_**^.^**^-^) is the precursor of hydrogen peroxide (H_2_O_2_), which can be consumed by myeloperoxidase (MPO) to produce the very potent bactericidal and oxidizing compound hypochlorous acid (HOCl). Finally, superoxide can directly react with nitric oxide (NO**^.^**) to form the highly reactive peroxynitrite (ONOO**^.^**^-^) 4.

In addition to their antimicrobial properties, ROS/RNS can activate the nuclear factor erythroid 2-related factor 2 (NRF2) stress response and mitogen-activated protein (MAP) kinase pathway or interact with the NF-κB signaling pathway. Therefore, ROS/RNS are able to influence the expression of various proinflammatory cytokines 7-11. In contrast, excessive levels of ROS/RNS can oxidize proteins, lipids and DNA, leading to serious cellular injury and contributing to the pathogenesis of several diseases 12, 13.

Furthermore, ROS and RNS contribute to the formation of neutrophil extracellular traps (NETs), which are web-like chromatin structures resulting from intracellular protein release by PMNs 14-17. NETs not only are capable of trapping invading pathogens but also affect many aspects of innate and adaptive immunity 18, 19. Overall, ROS and RNS can modulate the immune response in various ways, making these species attractive therapeutic targets in a broad range of diseases. Although many antioxidants exhibited promising results in experiments conducted both *in vitro* and in animals, most of them yielded no or only marginal therapeutic efficacy in clinical trials. No agent that primarily modifies antioxidant properties has become a standard of care treatment in the clinic 20-22.

We recently investigated the temporal dynamics of ROS/RNS production and NF-κB activation in an experimental model of 2,4,6-trinitrochlorobenzene (TNCB)-induced acute and chronic cutaneous DTHR, which is well suited for the investigation of the basic underlying mechanisms that drive T cell-mediated neutrophil-enriched autoimmune diseases 23.

Our studies revealed simultaneous peaks of ROS/RNS production and NF-κB activation during acute and chronic inflammation. The ROS/RNS peaks correlated well with PMN accumulation during acute and chronic cutaneous DTHR 23. However, the impact of various ROS/RNS sources on the development of different stages of T cell-driven neutrophil-enriched cutaneous immune responses has not been sufficiently characterized to date.

Therefore, we compared *in vivo* and *ex vivo* ROS/RNS production, biochemical and morphological characteristics and their clinical consequences in mice with differently impaired ROS/RNS production (MPO^-/-^, NOX2^-/-^, and iNOS^-/-^ mice) to those in wild-type mice during T cell-dependent acute and chronic TNCB-induced cutaneous DTHR (Figure S1).

## Materials and Methods

### Animals

Breeding pairs of C57BL/6J, B6.129S-Cybb^tm1Din^/J (gp91^phox-/-^), B6.129X1-Mpo^tm1Lus^/J (MPO^-/-^) and B6.129P2-Nos2^tm1Lau^/J (iNOS^-/-^) mice were purchased from Jackson Laboratory (Bar Harbor, USA). We used 8- to 12-week-old female mice for our *in vivo* experiments, and these animals were all bred and raised under the same conditions in the central animal housing facility of the University of Tübingen. B6(Cg)-Padi4^tm1.2Kmow^/J mice (PAD4^-/-^) were kindly provided by Prof. Wolfram Ruf, University of Mainz. All the experimental protocols were approved by Regierungspräsidium Tübingen.

### Cutaneous delayed-type hypersensitivity reaction (DTHR)

Wild-type, gp91^phox-/-^, MPO^-/-^, iNOS^-/-^ or PAD4^-/-^ mice were sensitized with 5 % TNCB at the abdomen and challenged after one week on the right ear with 1 % TNCB to elicit acute DTHR (n = 11-12). Chronic DTHR was induced by repetitive challenges with 1 % TNCB every 2-3 days for up to five times (1^st^ TNCB challenge: day 0; 2^nd^: day 3; 3^rd^: day 5; 4^th^: day 8, 5^th^: day 10). To monitor the course of the ear swelling responses, the mice were anesthetized by inhalation of isoflurane-O_2_ (1.5 % Forane, Abbott GmbH, Wiesbaden, Germany), and the ear thickness was measured with a micrometer (Kroeplin, Schlüchtern, Germany) before and 4 h, 12 h and 24 h after each TNCB challenge. Moderate cutaneous DTHR was established by reducing the concentration of the TNCB challenge solution from 1 % to 0.5 % following the same protocol (Figure S1).

### Treatment approach

Gp91^phox-/-^ mice were treated daily with MitoTEMPO (1.5 mg/kg; Sigma Aldrich, Germany) or were sham-treated with NaCl solution *i.p*., starting three days before the first TNCB challenge continuing until the 5^th^ TNCB challenge.

### *In vivo* optical imaging

For the *in vivo* ROS/RNS measurements, we used the ROS/RNS-sensitive chemiluminescent probe L-012 (Wako Chemical, Neuss, Germany). L-012 was dissolved in ultrapure H_2_O (5 mg/mL) and injected intraperitoneally (100 µL) 5 min before the OI measurement. For the *in vivo* investigations, we used the IVIS Spectrum OI System (Perkin Elmer, Rodgau-Jügesheim, Germany). Wild-type, gp91^phox-/-^, MPO^-/-^, iNOS^-/-^ and PAD4^-/-^ mice were anesthetized by inhalation of isoflurane-O_2_ (1.5 % Forane) and warmed on a heating pad to maintain a body temperature of 37° C. OI was performed before and 4 h, 12 h and 24 h after the 1^st^, 3^rd^ and 5^th^ TNCB challenges (n = 11-12 per group). The images were analyzed with the Living Image software (Perkin Elmer, Waltham, USA) by drawing regions of interest on the left and right ear, allowing a semi-quantitative analysis of the average radiance (photons per second per square centimeter per steradian; p/s/cm^2^/sr) emitted by the chemiluminescent probe L-012.

### DHR flow cytometry analysis

Cells from the inflamed ear tissue (n = 5) and the draining lymph nodes (dLNs; n = 5) were isolated from wild-type, gp91^phox-/-^, MPO^-/-^ or iNOS^-/-^ mice with acute DTHR (24 h after the 1^st^ challenge). Intracellular ROS/RNS levels were evaluated by a dihydrorhodamine 123 (DHR) flow cytometry assay (Sigma Aldrich, St. Louis, USA) as described elsewhere 24. Briefly, to determine the ROS/RNS intermediates, the dLNs or ear tissues were digested in RPMI medium containing collagenase IV, DNase, HEPES and fetal bovine serum and passed through a 70 µm and a 40 µm cell strainer to obtain a single cell suspension. The cells were stimulated for 30 min with 0.5 µg/mL phorbol-12-myristat-13-acetate (PMA) at 37 °C and stained with 30 µg/mL DHR. The fluorescence signal intensity (FI) was quantified by flow cytometry (LSR Fortessa; BD Biosciences, Franklin Lakes, USA) and analyzed using FlowJo (FlowJo, LLC, Ashland, USA).

### Lipid peroxidation assay

Oxidative stress in inflamed ears of wild-type, gp91^phox-/-^, MPO^-/-^ and iNOS^-/-^ mice with chronic DTHR (24 h after the 5^th^ challenge, n = 5 per group) was quantified by assessing lipid peroxidation. Lipid peroxidation was assessed by measuring malondialdehyde (MDA) in ear tissue lysates using a commercial assay kit (Sigma-Aldrich, St. Louis, USA) as described by the manufacturer.

### Oxidative DNA damage assay

Genomic DNA was extracted from ears tissue of healthy wild-type mice (naïve) and wild-type, gp91^phox-/-^, MPO^-/-^ and iNOS^-/-^ mice with acute or chronic cutaneous DTHR (24 h after the 1^st^ and 5^th^ TNCB ear challenge, n = 4 per group) using the NucleoSpin Tissue Kit (Takara Bio Inc, Kusatsu, Japan). Extracted DNA was digested with DNase I (Sigma) and S1 Nuclease (Thermo Fisher, Waltham, USA) following by incubation with alkaline phosphatase (Thermo Fischer) according to the protocol by Huang et al. 25. 8-hydroxy-2'-deoxyguanosine (8-OHdG) levels were determined with DNA/RNA Oxidative Damage ELISA Kit (Cayman, Ann Arbor, USA).

### Histopathology and immunohistochemistry

Representative inflamed ears from wild-type, gp91^phox-/-^, MPO^-/-^, iNOS^-/-^ or PAD4^-/-^ mice with acute or chronic cutaneous DTHR (24 h after the 1^st^ and 5^th^ TNCB ear challenge, n = 4 per group) were collected. After fixation in 4 % formalin, the tissue was dehydrated, embedded in paraffin, cut into 3-5 μm sections and stained with hematoxylin and eosin (H&E). Immunohistochemistry (IHC) was performed with antibodies (Ab) against CD3 (clone SP7, DCS Innovative Diagnostik-Systeme GmbH u. Co. KG, Hamburg, Germany) and MPO (anti-MPO Ab-1, Lab Vision UK, Ltd., Newmarket, Suffolk, Great Britain) on an automated immunostainer (Ventana Medical Systems, Inc.) according to the company's protocols for open procedures with slight modifications. A Zeiss Axioskop 2 plus microscope equipped with a Jenoptik ProgRes C10 plus camera and software (Laser Optik System, Jena, Germany) was used to acquire the photomicrographic images. The epidermal inflammation score was determined by counting the epidermal abscesses and crusts per section (0 = no damage, 1 = presence of abscesses, 2 = between 1 and 5 crusts, 3 = between 6 and 10 crusts, 4 = more than 11 crusts). The following score was used to evaluate PMN and T cell infiltration in MPO and CD3 immunohistochemical analysis: “0” = no inflammatory cell infiltrate, “1” = minimal inflammatory cell infiltrate, “2” = mild inflammatory cell infiltrate, “3” = moderate inflammatory cell infiltrate, “4” = severe and dense inflammatory cell infiltrate. The quantification of the PMNs in MPO^-/-^ mice was determined morphologically.

### Fluorescence Microscopy

Inflamed ears of wild-type and gp91^phox-/-^ mice with acute (7 h and 24 h after the 1^st^ TNCB ear challenge, n = 3) or chronic (7 h after the 5^th^ TNCB ear challenge, n = 2-3) cutaneous DTHR and inflamed ears of PAD4^-/-^, MPO^-/-^ and iNOS^-/-^ mice with acute (7 h after 1^st^ TNCB challenge; n = 3) cutaneous DTHR were immediately placed in RPMI (Biochrom, Berlin, Germany) and frozen in liquid nitrogen. The ears that were frozen in RPMI were cut in 5 µm sections and fixed with periodate-lysine-paraformaldehyde (0.1 M L-lysine-HCl, 2 % paraformaldehyde, 0.01 M sodium metaperiodate pH 7.4) and blocked using donkey serum at a 1:20 dilution. The sections were incubated with the following Ab: rabbit anti-OGG1 Ab 1:50 (Novus Biologicals, Centennial, USA), rabbit anti-TOMM20 Ab 1:50 (Novus Biologicals, Centennial, USA), rat anti-F4/80 Ab 1:50 (eBioscience, San Diego, USA), rabbit anti-Elastase 1:50 (Abcam, Cambridge, UK), goat anti-H2A.X Ab 1:100 (Abcam), rabbit anti-H3 cit. 1:100 (Abcam), and goat anti-MPO Ab 1:50 (R&D Systems, Minneapolis, USA). The bound Abs were visualized using Alexa 488-donkey anti-goat Ab 1:250 (Dianova, Hamburg, Germany), Cy3-donkey anti-rabbit Ab 1:250 (Dianova), Cy3-donkey anti-rat Ab (Dianova), Cy3-donkey anti-goat Ab (Dianova) and Alexa 488-donkey anti-rabbit Ab (Dianova). Nuclear staining was performed with DAPI 1:10000 (Sigma-Aldrich, St. Louis, USA). The slides were mounted using Mowiol (Hoechst, Frankfurt, Germany) and analyzed using a Zeiss LSM800 confocal laser scanning microscope (Zeiss, Oberkochen, Germany) and images were processed with the software ZEN 2.3 (blue edition) and the Image Analysis Module (Zeiss) or a Leica TCS-SP/Leica DM RB confocal laser scanning microscope (Leica Microsystems, Wetzlar, Germany). Processing of the images was performed with Leica Confocal Software LCS (Version 2.61). NET formation was semi-quantitatively analyzed by determining the area [µm^2^] of OGG1 staining outside the nuclei divided by the number of nuclei. OGG1 accumulation in the nucleus was analyzed by calculating the mean fluorescence intensity (MFI).

### RNA extraction and gene expression analysis

Frozen ear tissue from healthy wild-type mice (naïve) and wild-type, gp91^phox-/-^, MPO^-/-^ and iNOS^-/-^ mice with acute or chronic cutaneous DTHR (24 h after the 1^st^ and 5^th^ TNCB ear challenge, n = 4 per group) was homogenized in Qiazol (Qiagen, Hilden, Germany), and the total RNA was extracted according to the manufacturer's instructions.

DNase I digestion (EN0523, Thermo Fisher) was performed to remove the genomic DNA. RNA was reverse transcribed to cDNA using Revert Aid reverse transcriptase (EP0441, Thermo Fisher) and oligo(dT) primers (SO132, Thermo Fisher).

Real-time (RT) PCR was performed with Green Master mix (M3023, Genaxxon, Ulm Germany) and self-designed primers (Table S1). The samples were incubated for an initial denaturation for 15 min at 95 °C, followed by 45 cycles of 95 °C for 15 s and 60 °C for 45 s. The relative mRNA amount of each sample was calculated by normalization to the housekeeping gene *β-Actin* using the 2-ΔΔCT method.

### Flow cytometry analysis

The spleens and dLNs from wild-type, gp91^phox-/-^, MPO^-/-^ or iNOS^-/-^ mice with chronic cutaneous DTHR were isolated 24 h after the 5^th^ TNCB ear challenge, pressed through a 70 µm cell strainer to obtain a single cell suspension and washed in PBS with 1 % fetal bovine serum. Additionally, the erythrocytes in the cell homogenates from the spleen were lysed using ACK Lysing Buffer (BioWhittaker, Basel, Switzerland). The cells were counted with the C-Chip Disposable Counting Chamber (NanoEnTek, Seoul, Korea), and 5 x 10^6^ cells per sample were used for staining. The single cell suspensions were stained using the following mAbs: T cell panel: ZombieNIR-Viability, PE-CD45.2, AF700-CD8, FITC-CD3, BV510-CD44, BV650-CD69, BV711-PD-1, BV785-CD127, PE/Cy7-CD62L, PerCP-CD4, BV421-CD25 and FC-Block (BD Biosciences, Franklin Lakes, USA); myeloid cell panel: PE/Cy7-Ly6cC, ZombieNIR-Viability, BV-605-PD-L1, FITC-Ly6G, PerCP-MHCII, APC-CD11c, BV510-CD11b, BV650-CD69, BV785-B220, BV421-CD3, PE-NKp46, and FC-Block (BD Biosciences). Single-cell suspensions were measured on a BD LSR Fortessa (BD Biosciences) flow cytometer and analyzed using FlowJo software (FlowJo, Ashland, USA). The gating strategy for the T cells and myeloid cells is represented in Figure S2.

### Metabolomics

Deep-frozen ear samples with chronic cutaneous DTHR (4 h after 5^th^ challenge) were cryogenically pulverized (Covaris cryoPREP CP02, Woburn, USA) and the homogenous tissue powder suspended in 300 µL of ultrapure methanol and 1000 µL of *tert*-butyl methyl ether (MTBE). Metabolites were extracted with Adaptive Focused Acoustics (AFA^TM^) using a 5 min ultrasonication program per sample (Covaris E220 Evolution, Woburn, USA). After extraction, 250 µL of ultrapure water was added for phase separation and the polar phase was evaporated to dryness overnight. The pellets were resuspended in a deuterated phosphate buffer (pH 7.4) containing 1 mM of internal quantification standard. NMR spectra were recorded on a 600 MHz (14.10) Tesla ultra-shielded NMR spectrometer (Avance III HD, Bruker BioSpin, Karlsruhe, Germany) with a triple resonance 1.7 mm room temperature probe at 298 K. Depending on signal-to-noise ratio 1024 or 2048 scans were performed per measurement using a water suppression pulse program. Metabolites were annotated and quantified employing a commercial database (ChenomX NMR Suite 8.3).

### Statistical analysis

Statistical analyses and graph design were performed with GraphPad Prism (Version 7.03, GraphPad Software, Inc., USA). The Kruskal-Wallis test with post hoc Dunn's test was used for the analysis of OI SIs, ear swelling response differences, lipid peroxidation, cell population flow cytometry analysis, DHR flow cytometry analysis and mRNA expression by comparing the wild-type group with the gp91^phox-/-^, MPO^-/-^ and iNOS^-/-^ groups as well as for NET formation and nuclear OGG1 accumulation by comparing the wild-type group with the gp91^phox-/-^ and PAD4^-/-^ groups (p-values were adjusted for multiple comparisons).

Unpaired, two-tailed Student's t-test was used to compare the differences in the ear swelling responses between the sham- and MitoTEMPO-treated groups and between the wild-type and PAD4^-/-^ mice. OI SI from wild-type and PAD4^-/-^ mice were compared using the Mann-Whitney test.

Metabolomics data was statistically analyzed using the MetaboAnalyst 4.0 web server. Metabolite concentrations were normalized using the percentile quantile normalization (PQN) approach to account for dilution effects and then pareto-scaled 26, 27 prior to statistical analysis, including two dimensional principal component analysis (PCA) and hierarchical cluster analysis (HCA).

## Results

### Dynamics of* in vivo* ROS and RNS production during acute and chronic cutaneous DTHR

**Figure 1:**
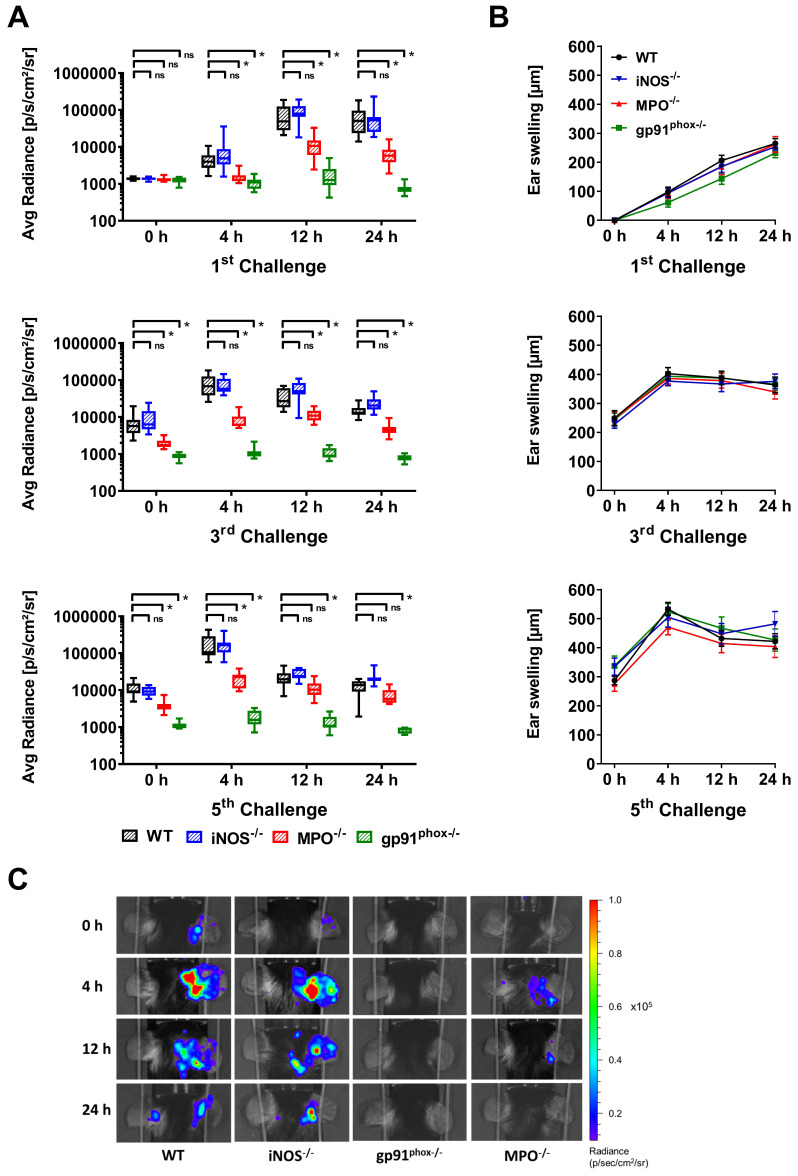
***In vivo* optical imaging of ROS/RNS production and ear swelling response. (A)** *In vivo* ROS/RNS production in the acute (1^st^ challenge), early chronic (3^rd^ challenge) and chronic (5^th^ challenge) cutaneous DTHR. L-012 was injected into WT (n=12), gp91^phox-/-^ (n=11), MPO^-/-^ (n=11) and iNOS^-/-^ mice (n=12) 5 min before the optical imaging measurement. ROS/RNS levels are displayed as the medians with interquartile ranges of average radiance (p/s/cm^2^/sr); whiskers indicate the min and max values. **(B)** Ear swelling responses in the acute, early chronic and chronic DTHR. Data are displayed as mean ± SEM. No significant differences between WT mice (n=12) and gp91^phox-/-^ (n=11), MPO^-/-^ (n=11), or iNOS^-/-^ (n=12) mice were observed (Kruskal-Wallis test with post hoc Dunn test).** (C)** Representative optical imaging images of L-012 signal intensity in each experimental group after the 5^th^ TNCB challenge.

First, we noninvasively examined ROS/RNS production *in vivo* in wild-type, gp91^phox-/-^, MPO^-/-^ and iNOS^-/-^ mice by OI at various time points of acute and chronic inflammation by employing L-012, a well-established, luminol-based chemiluminescent probe. Thus, we investigated experimental mice with acute (1^st^ challenge), early chronic (3^rd^ challenge) and chronic (5^th^ challenge) cutaneous DTHR (Figure S1). The OI measurements revealed a strongly enhanced L-012 SI in the inflamed ears of wild-type mice 12 h and 24 h after the 1^st^ TNCB ear challenge, indicating drastically elevated ROS/RNS levels during the acute phase of inflammation. In contrast, ROS/RNS production during the early chronic and chronic DTHR peaked at 4 h after the 3^rd^ and 5^th^ TNCB ear challenges (Figure 1A). In comparison to those of wild-type mice, the inflamed ears of gp91^phox-/-^ mice exhibited completely abrogated ROS/RNS production during the acute, early chronic and chronic DTHR, since the L-012 SI remained at baseline levels at all the investigated time points (Figure 1A, p < 0.05). However, the inflamed ears of MPO^-/-^ mice with acute, early chronic and chronic DTHR exhibited an up to 90 % decrease in L-012 SI compared to the inflamed ears of wild-type mice (Figure 1A; p < 0.05). In contrast, we observed no significant differences in L-012 SI in the inflamed ears of iNOS^-/-^ mice with acute, early chronic and chronic DTHR when compared to the inflamed ears of wild-type mice (Figure 1A).

Simultaneously to the OI measurements, we determined ear swelling responses as a clinical indicator of the severity of the DTHR. The ear swelling responses and ROS/RNS production peaked simultaneously in the early chronic and chronic DTHR in wild-type, MPO^-/-^ and iNOS^-/-^ mice (Figure 1A-B). A statistically significant correlation was found between the increase in ear swelling and the increase in L-012 SI in wild-type mice (r = 0.79, p = 0.0117; Figure S3). Surprisingly, we observed no significant differences in the ear swelling responses between wild-type and gp91^phox-/-^, MPO^-/-^ and iNOS^-/-^ mice at all investigated time points (Figure 1B). The strongly reduced ROS/RNS (Figure 1A, C) production in gp91^phox-/-^ and MPO^-/-^ mice had no effect on the ear swelling response (Figure 1B).

Additionally, flow cytometry analysis of the splenic and lymph node-derived lymphocytes revealed no significant differences in the expression of the T cell activation marker CD69 and the immune checkpoint PD-1 among the experimental groups (Figure S4). These observations indicated a normal sensitization phase with effective T cell priming. Notably, iNOS^-/-^ mice exhibited a significantly increased monocyte population in the spleen and a decreased number of naïve CD8^+^ T cells in the dLNs (Figure S4).

Ear challenges with a 1 % TNCB solution might elicit a severe inflammatory immune response that could overcome the immune modulatory effect of ROS/RNS in inflammation. Therefore, we used a 0.5 % TNCB challenge solution to elicit a less-pronounced inflammatory immune response. Consequently, we observed reduced ear swelling responses accompanied by reduced ROS/RNS production (except at 4 h after the 5^th^ challenge) in the wild-type group and the other experimental groups (Figure S5). However, we again observed no significant differences in the ear swelling responses between the different mouse models of impaired ROS/RNS production and the wild-type mice (Figure S5).

### *Ex vivo* cross correlation of the *in vivo* results of ROS/RNS production

**Figure 2:**
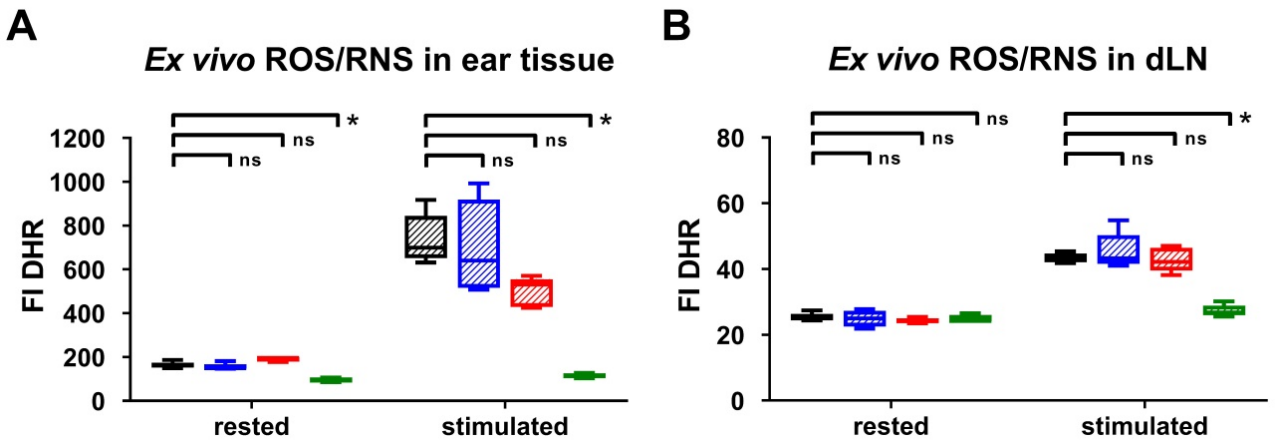
***Ex vivo* intracellular ROS/RNS analysis.** *Ex vivo* dihydrorhodamine 123 (DHR) flow cytometry analysis of ROS/RNS stress in inflamed ear tissue **(A)** and draining lymph nodes (dLN) **(B)** 24 h after the 1^st^ challenge. Rested cells were incubated with 30 µg/mL DHR. Stimulated cells were treated with 0.5 µM phorbol-12-myristat-13-acetate (PMA) prior to incubation with 30 µg/mL DHR. FI: fluorescence intensity. Data are displayed as the medians with interquartile ranges; whiskers indicate the min and max values, *p < 0.05, ns = not significant (Kruskal-Wallis test with post hoc Dunn test; p-values corrected for multiple comparisons n = 5 in each group).

To validate our *in vivo* OI results, we performed *ex vivo* DHR flow cytometry analysis of the ROS/RNS production in the inflamed ears and dLNs 24 h after the 1^st^ TNCB-ear challenge. Unstimulated cells derived from the inflamed ears of gp91^phox-/-^ mice exhibited up to 50 % lower ROS/RNS production (median FI: 97 (interquartile ranges IR: 89 - 102)) compared to the inflamed ears of iNOS^-/-^ (FI: 150 (IR:147 - 167)), MPO^-/-^ (FI: 196 (IR: 200 - 179)) and wild-type mice (FI: 161 (IR: 154 - 175)) (Figure 2A). Stimulation with PMA caused a pronounced increase in the ROS/RNS production in the cells derived from the inflamed ears and dLNs of wild-type, iNOS^-/-^ and MPO^-/-^ mice, while the ROS/RNS production in the cells derived from the inflamed ears of gp91^phox-/-^ mice was slightly elevated (FI: 113 (IR: 105 - 127)) in comparison to the unstimulated cells (Figure 2A-B). Stimulated cells derived from the inflamed ears of iNOS^-/-^ mice exhibited similar ROS/RNS production levels (FI: 640 (IR: 515 - 919)) when compared to those of wild-type mice (FI: 700 (IR: 652 - 845)) (Figure 2A). In contrast, the ROS/RNS production in the cells derived from the inflamed ears of MPO^-/-^ mice (FI: 531 (IR: 429 - 555)) decreased by approximately 25 % in comparison to that in the cells derived from wild-type mice (Figure 2A). A significant reduction in the FI of the PMA-stimulated cells derived from the inflamed ears and dLNs of gp91^phox-/-^ and wild-type mice was observed (p < 0.05; Figure 2A-B). Thus, the flow cytometry analysis clearly confirmed our *in vivo* OI analysis, since both methods displayed low levels or no expression of ROS/RNS in the inflamed ears and dLNs of gp91^phox-/-^ mice, decreased levels in the inflamed ears of MPO^-/-^ mice and similar levels in the inflamed ears of iNOS^-/-^ mice compared to the levels in the inflamed ears of wild-type mice. Taken together, these data indicate that NOX2 and MPO are the dominant sources of ROS production during cutaneous DTHR.

### Histopathological evaluation of the consequences of ROS/RNS deficiency

To better characterize the inflammatory immune response, we obtained inflamed ears from wild-type, gp91^phox-/-^, MPO^-/-^ and iNOS^-/-^ mice 24 h after the 1^st^ (acute DTHR) and 5^th^ (chronic DTHR) TNCB ear challenges and conducted H&E as well as CD3 and MPO IHC. Despite the similar ear swelling responses during the acute DTHR, the inflamed ears from gp91^phox-/-^ mice exhibited a more severe inflammatory immune response.

**Figure 3:**
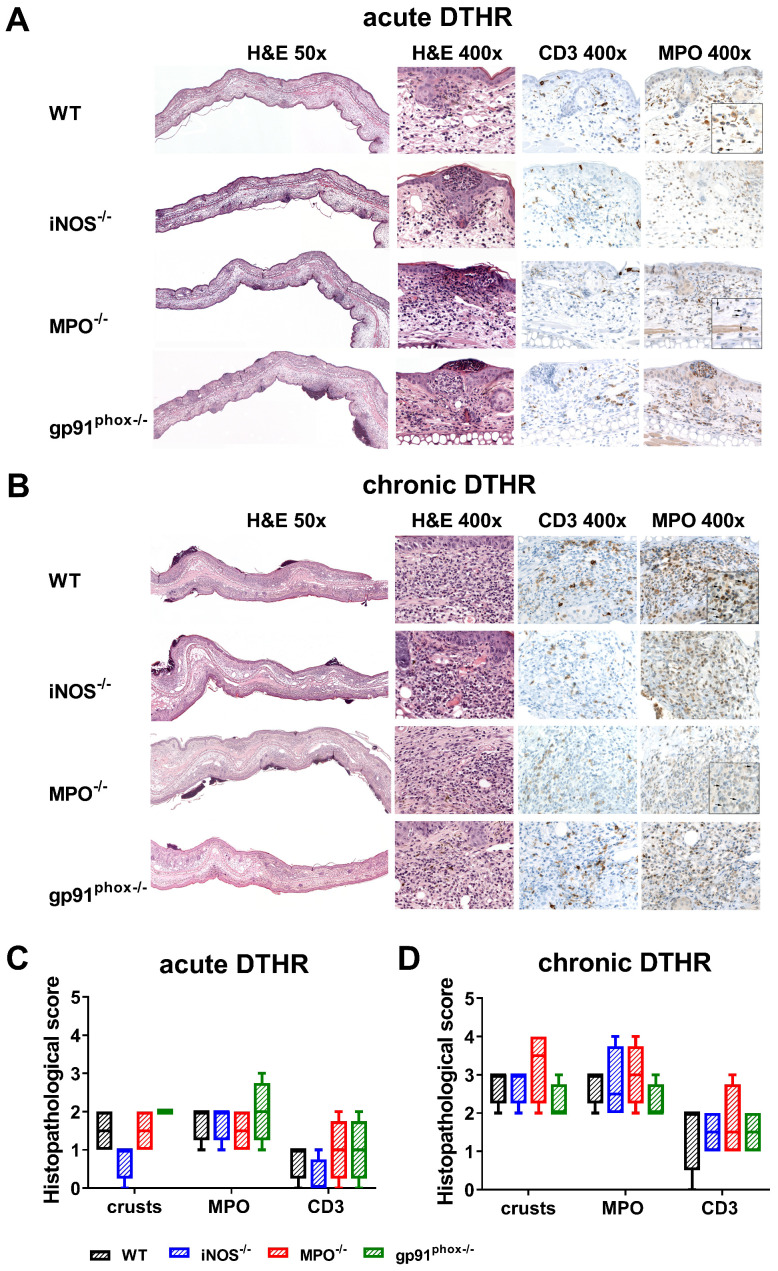
** Histopathological analysis of acute and chronic DTHR.** Representative images of inflamed ear tissue with **(A)** acute DTHR (24 h after the 1^st^ challenge) and **(B)** chronic DTHR (24 h after the 5^th^ challenge). Insert with increased magnification of the MPO immunohistochemistry of inflamed ears with acute and chronic cutaneous DTHR derived from wild-type and the MPO^-/-^ mice that clearly highlight the morphology of the segmented neutrophils (indicated by arrows) and the positive staining in wild-type but not in MPO^-/-^ mice. Some brown staining, corresponds partially to melanin pigment that is present in macrophages. Additionally, some non-specific pale brown stain can also be observed in the cytoplasm of histiocytes. Histopathological analysis of **(C)** acute and **(D)** chronic DTHR. Histopathological score was assessed by number of epidermal abscesses and crusts per section (0 = no crusts or abscesses; 1 = abscesses, no crusts; 2 = between 1 and 5 crusts, 3 = between 6 and 10 crusts, 4 = more than 11 crusts). Neutrophil (MPO) abundance and T cell (CD3) abundance were determined by a semi-quantitative analysis of dermal inflammation (0 = no inflammatory infiltrate; 1 = minimal inflammatory infiltrate; 2 = mild inflammatory infiltrate; 3 = moderate inflammatory infiltrate; and 4 = severe inflammatory infiltrate). Quantification of the neutrophils in MPO^-/-^ mice was determined morphologically. Data are displayed as the medians with interquartile ranges; whiskers indicate the min and max values (n = 4 for each experimental group).

Histopathological analysis revealed multiple lesions, crusts, some ulceration of the epidermis and more abundant inflammatory infiltrate in the dermis in comparison to those of the inflamed ears of MPO^-/-^, iNOS^-/-^ and wild-type mice, where the infiltrates were mostly limited to the epidermis (Figure 3A, C). IHC confirmed an enhanced abundance of T cells and PMNs in the epidermis and dermis in the inflamed ears of gp91^phox-/-^ mice compared to those of the other experimental groups (Figure 3C). In contrast, during chronic DTHR, the inflamed ear tissue of gp91^phox-/-^ mice exhibited less inflammation with reduced edema and PMN infiltrate compared to the tissue of wild-type and iNOS^-/-^ mice. Inflamed ears of MPO^-/-^ mice exhibited the most affected phenotype, with severe damages of the epidermis, deep ulcers and PMN abscesses (Figure 3B, D).

### Impact of ROS/RNS deficiency on the genomic expression of antioxidant proteins, proinflammatory cytokines and chemokines

**Figure 4:**
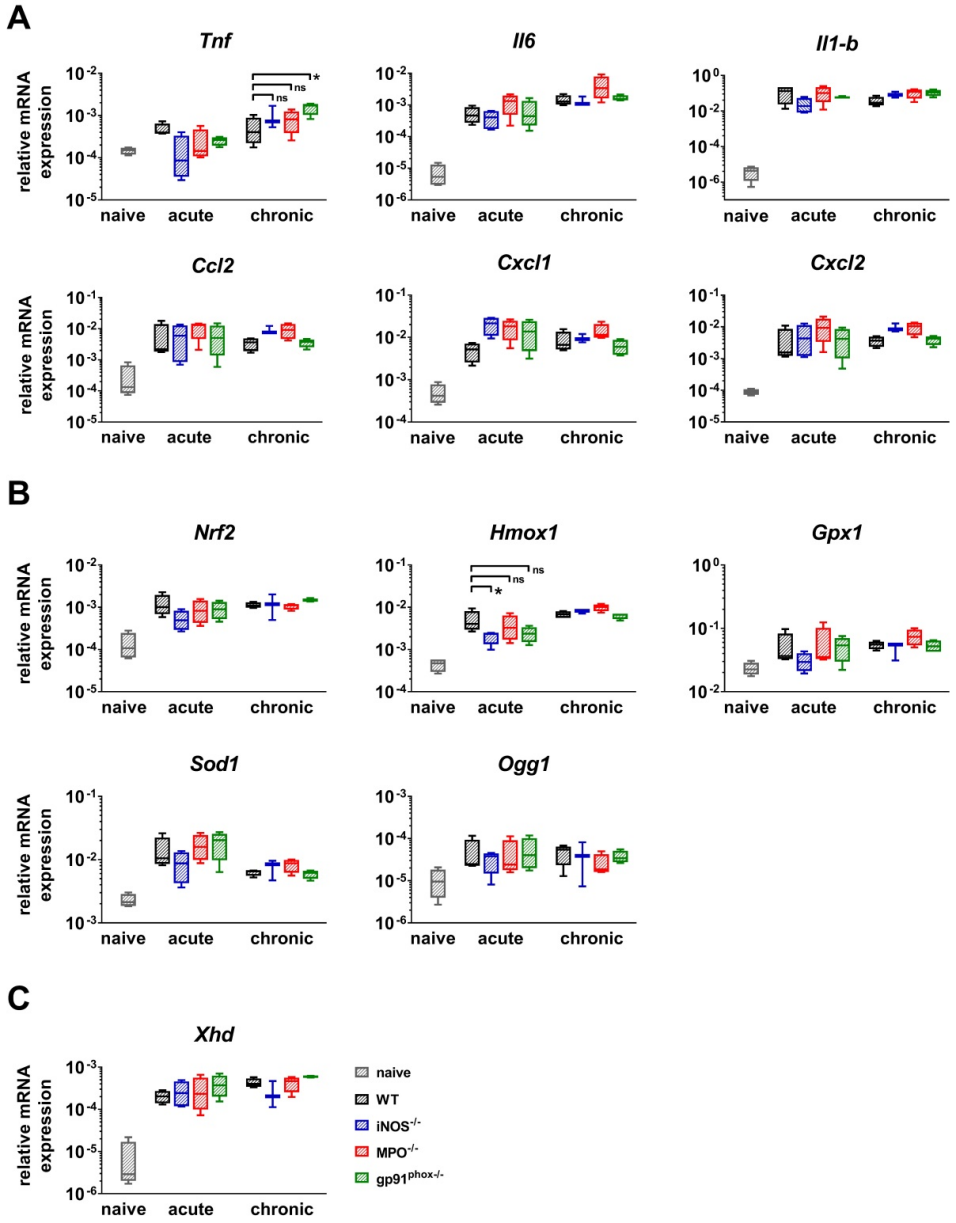
** RT-PCR analysis of acute and chronic DTHR.** Inflamed ear tissue was harvested 24 h after the 1^st^ TNCB challenge (acute DTHR) and 4 h after the 5^th^ challenge (chronic DTHR). The ear tissue of naïve wild-type mice was used as a control. **(A)** mRNA expression of NF-κB regulated proinflammatory cytokines and chemokines. **(B)** mRNA expression of redox-related proteins. **(C)** mRNA expression of xanthine oxidase (*Xhd*). Data are displayed as the medians with interquartile ranges; whiskers indicate the min and max values (n = 4). *p < 0.05, ns = not significant (Kruskal-Wallis test with post hoc Dunn test; p-values corrected for multiple comparisons).

Furthermore, we analyzed the gene expression of NF-κB-regulated inflammatory cytokines (*Il-6*, *Il-1b* and *Tnf*) and chemokines (*Cxcl1*, *Cxcl2* and *Ccl2)* that are responsible for the recruitment of PMNs, T cells and DCs 28-31. We analyzed healthy and inflamed ears from wild-type mice and inflamed ears from gp91^phox-/-^, MPO^-/-^ and iNOS^-/-^ mice. The inflamed ears were harvested at the time points with the strongest ear swelling responses during the acute (24 h after the 1^st^ challenge) and chronic (4 h after the 5^th^ challenge) DTHR (Figure 1B). Inflamed ears with acute cutaneous DTHR from gp91^phox-/-^, MPO^-/-^ and iNOS^-/-^ mice displayed a tendency towards an increase in the *Cxcl1* mRNA expression and a decrease in the *Tnf* mRNA expression levels when compared to wild-type mice (Figure 4A). During chronic DTHR, the *Tnf* mRNA expression in the inflamed ears of gp91^phox-/-^ mice was significantly enhanced (Figure 4A) when compared to wild-type mice, which was inconsistent with the reduced inflammatory immune response observed by histopathology (Figure 3B, D). In addition, we determined a tendency towards an increased expression of *Il-6*, *Ccl2, Cxcl1* and *Cxcl2* mRNA in inflamed ears of MPO^-/-^ mice with chronic cutaneous DTHR when compared to wild-type mice (Figure 4A).

Next, we focused on investigating the mRNA expression patterns of the transcription factor *Nrf2*, which regulates the expression of various antioxidant proteins such as *Hmox1*, *Gpx1* and *Sod1*. In addition, we analyzed the mRNA expression of 8-hydroxy-2'deoxyguanosine DNA glycosylase1 (*Ogg1*), an enzyme that is responsible for the excision of 8-OHdG, a mutagenic base that is formed by exposure of DNA to ROS/RNS 32. Our RT-PCR analysis revealed a strong upregulation of *Nrf2*, *Gpx1*, *Sod1* and *Ogg1* mRNA expression in all experimental groups during acute and chronic DTHR. However, only *Hmox1* expression was significantly reduced in inflamed ears of iNOS^-/-^ mice during acute DTHR when compared to inflamed ears of wild-type mice. Further, we compared the mRNA expression levels of different ROS/RNS producing proteins. We observed a strong upregulation of xanthine oxidase (*Xhd*) in all experimental groups during acute and chronic DTHR (Figure 4C), while the mRNA expression of *iNos*, *eNos*, *Mpo* and *Nox4* was below the detection limit of 32 cycles and therefore negligible (data not shown).

### Impact of ROS/RNS deficiency on oxidative DNA damage and lipid peroxidation and metabolism

**Figure 5:**
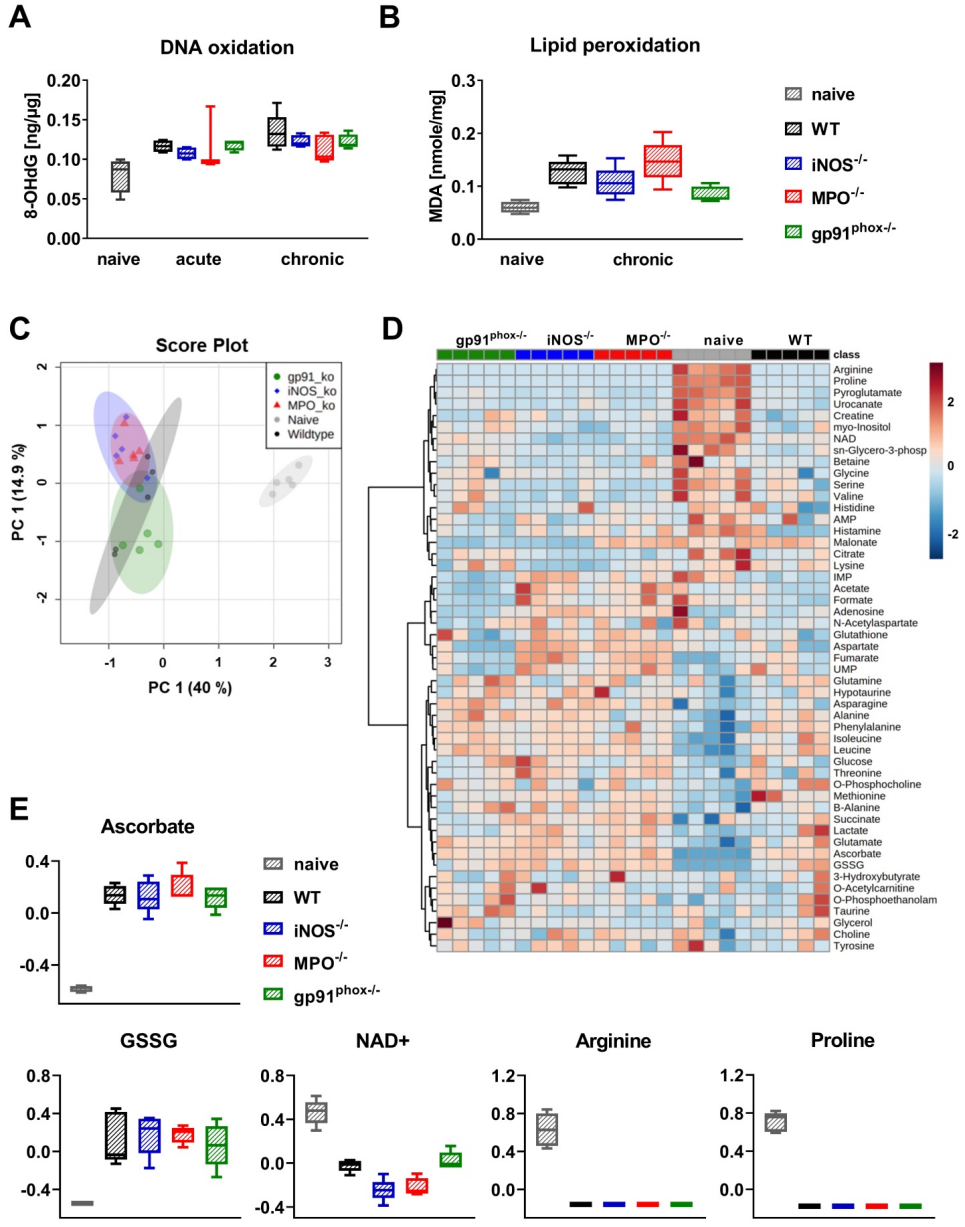
** Consequences of ROS/RNS deficiency on DNA oxidation, lipid peroxidation and metabolism.** Ear tissue samples of WT, iNOS^-/-^, MPO^-/-^ and gp91^phox-/-^ mice (n = 5) were collected 24 h after the 1^st^ and 5^th^ TNCB challenge. As a control, we used ear tissue from untreated naïve mice (n = 4 - 5). **(A)** Oxidative DNA damage in ear tissue with acute and chronic DTHR. 8-hydroxy-2'deoxyguanosine (8-OHdG) ng/µg DNA. **(B)** Lipid peroxidation in chronic DTHR. Malondialdehyde (MDA) in nmol/mg ear tissue. **(C)** Principal component analysis (PCA) of the normalized metabolite data of inflamed ears with chronic DTHR. Shaded areas represent respective 95 % confidence intervals of the 5 clustered groups (naïve WT, WT, iNOS^-/-^, MPO^-/-^ and gp91^phox-/-^ mice). **(D)** Hierarchical clustering analysis (HCA) and heatmap of the 48 analyzed metabolites. Color scale illustrates the abundance of each metabolite. Red: high abundance, blue: low abundance. **(E)** Difference in redox-related metabolites; ascorbate, glutathione disulfide (GSSG), nicotinamide adenine dinucleotide (NAD^+^), arginine and proline. Data are expressed as the medians with interquartile ranges; whiskers indicate the min and max values. No significant difference between the WT and other experimental groups was observed (Kruskal-Wallis test with post hoc Dunn test).

Since the OI and DHR flow cytometry analysis revealed abrogated ROS/RNS production in gp91^phox-/-^ mice, we were interested in whether this reduced ROS/RNS production caused a decreased oxidative damage and metabolic changes in the inflamed ear tissue of mice with acute and chronic cutaneous DTHR. To address this question, we examined 8-OHdG levels for DNA oxidation and malondialdehyde (MDA) levels for lipid peroxidation as markers of oxidative stress in the inflamed ears of wild-type, gp91^phox-/-^, MPO^-/-^ and iNOS^-/-^ mice. All experimental groups exhibited higher levels of 8-OHdG in acute and chronic DTHR. To our surprise, we detected no significant differences in DNA oxidation between inflamed ears of wild-type mice and the inflamed ears of the ROS/RNS-deficient mice (Figure 5A). Analysis of lipid peroxidation during chronic DTHR revealed similar results (Figure 5B). Nonetheless, we observed a tendency towards a reduced concentration of lipid peroxides in inflamed ears of gp91^phox-/-^ mice when compared to ears of wild-type mice (p = 0.065: not significant; Figure 5B).

Next, we performed ^1^H NMR-based metabolomics to identify metabolic changes in inflamed ears of wild-type, gp91^phox-/-^, MPO^-/-^ and iNOS^-/-^ mice with chronic DTHR. Herein, a principal component analysis (PCA) of the normalized metabolite data revealed a very distinct clustering of all experimental groups with chronic DTHR from non-inflamed ears of naïve mice along the principal component 1 (PC1) dimension (Figure 5C). By contrast, only a moderate separation of the metabolic profiles was observed in the inflamed ears with chronic DTHR of all ROS/RNS deficient mice when compared to the ears of wild-type mice, likely as a consequence of the substantial variation between individuals of the wild-type group (Figure 5C). Nevertheless, gp91^phox-/-^ mice were separated by their metabolic profile from the iNOS^-/-^ and MPO^-/-^ mice along the PC2 dimension. Next, the datasets of the 48 analyzed metabolites were used for hierarchical clustering analysis (HCA) and generation of a heatmap to visualize the clustering of all samples in each experimental group (Figure 5D). Focusing on redox-related metabolites, ascorbate, glutathione disulfide (GSSG), nicotinamide adenine dinucleotide (NAD^+^), arginine and proline revealed the most striking changes (Figure 5D, E). In detail, all experimental groups with chronic DTHR exhibited strongly elevated levels of ascorbate and GSSG in comparison to naïve mice. In sharp contrast, NAD^+^, arginine and proline levels were heavily depleted (Figure 5E). Taken together, DNA oxidation, lipid peroxidation, redox related metabolites and regulation of ROS/RNS driven genes indicate a similar level of oxidative stress in the inflamed ears of all experimental groups with chronic cutaneous DTHR.

Mitochondria are another source of ROS, which are capable of causing oxidative damage in cells and promoting chronic inflammatory diseases 33-36. Interestingly, daily treatment of gp91^phox-/-^ mice with 1.5 mg/kg MitoTEMPO, a ROS scavenger that specifically targets mitochondria 37 exhibited no therapeutic effects (Figure S7 A-C).

### OGG1 accumulation in NETs

**Figure 6:**
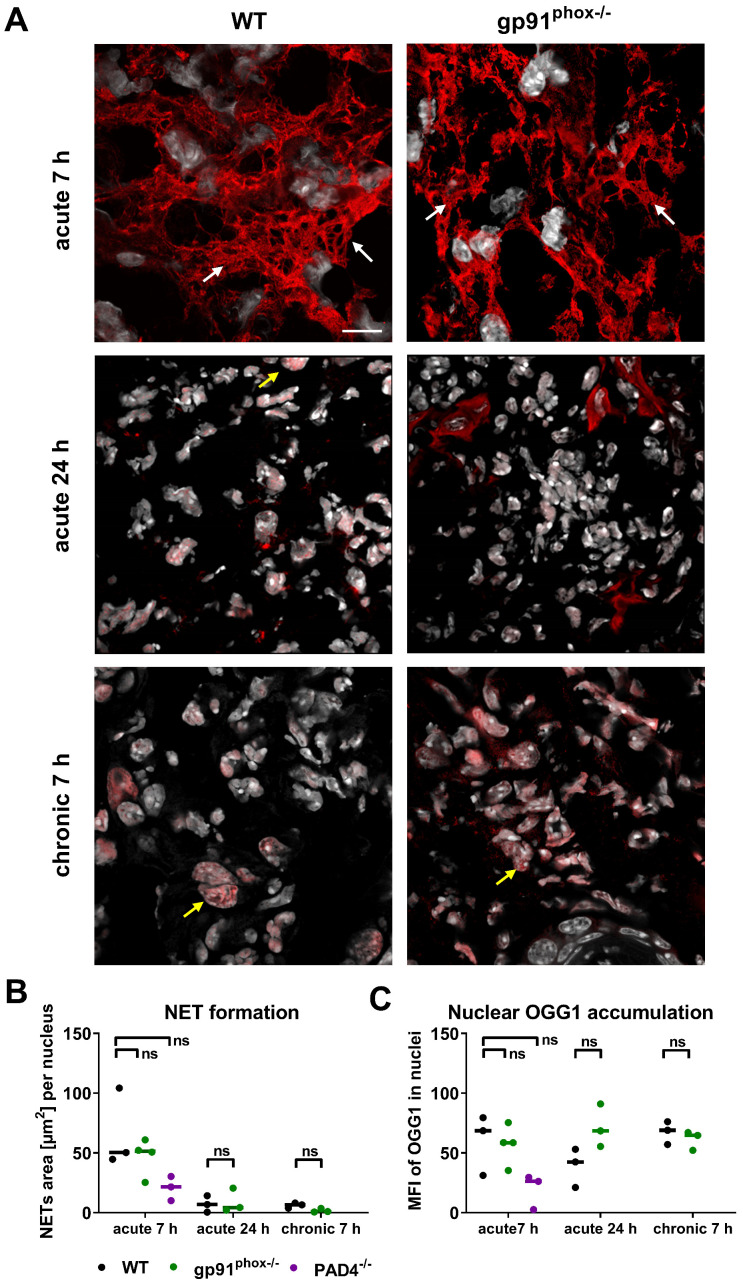
** NET formation and OGG1 accumulation in acute and chronic DTHR. (A)** Representative immunofluorescence images of the ear tissue of WT and gp91^phox-/-^ mice with acute 7 h/24 h and chronic 7 h DTHR. White arrows indicate NETs; yellow arrows indicate OGG1 accumulation in the nucleus. Red = OGG1, gray = DAPI, (n=3). The scale bar is 10 μm. **(B)** NET formation during acute 7 h and 24 h and chronic 7 h DTHR in WT, gp91^phox-/-^ and PAD4^-/-^ mice. NET formation was semi-quantitatively analyzed by determining the area [µm^2^] of OGG1 outside the nuclei divided by the number of nuclei. **(C)** OGG1 accumulation in the nucleus in acute 7 h and 24 h and chronic 7 h DTHR in WT, gp91^phox-/-^ and PAD4^-/-^ mice. OGG1 accumulation was semi-quantitatively analyzed by measuring the mean fluorescence intensity (MFI) in the nuclei. Horizontal lines indicate the medians. No significant difference among the wild-type, gp91^phox-/-^ and PAD4^-/-^ mice was observed (Kruskal-Wallis test with post hoc Dunn test; p-values corrected for multiple comparisons).

Recent studies reported that oxidative DNA damage and OGG1 expression is linked to proinflammatory immune responses 38, 39. Although the inflamed ears of gp91^phox-/-^ mice with acute and chronic DTHR displayed depletion of ROS/RNS production, levels of 8-OHdG and *Ogg1* mRNA expression were similar to wild-type mice. Therefore, we performed additional immunofluorescence stainings of OGG1 in inflamed ears of gp91^phox-/-^ and wild-type mice with acute and chronic DTHR. Both groups exhibited dense accumulation of OGG1-positive NET-like structures in the extracellular space as early as 7 h after the 1^st^ TNCB ear challenge (Figure 6A). NET formation was proven, as the NET-like structures in the inflamed ears of wild-type and gp91^phox-/-^ mice were close to MPO positive PMNs and stained positive for elastase and histone H2A.X but negative for TOMM20 (mitochondrial import receptor subunit) (Figure S7). This suggests that the NETs were released from the nucleus and not from mitochondria. In the inflamed ears of wild-type mice, we observed intense H3 citrullination, which was barely detectable in the ears of gp91^phox-/-^ mice (Figure S7). In contrast to the acute DTHR, we were not able to detect NETs in chronic DTHR (Figure 6A-B). In inflamed ears with acute cutaneous DTHR, OGG1 was predominantly present in the NETs and to a much lesser extent in the cell nuclei (Figure 6A). In line with wild-type and gp91^phox-/-^ mice, we observed NETs in inflamed ears of MPO^-/-^ and iNOS^-/-^ mice 7 h after the 1^st^ TNCB ear challenge (Figure S8). Semi-quantitative analysis of OGG1 expression in the nuclei revealed similar accumulation of OGG1 in inflamed ears of wild-type and gp91^phox-/-^ mice during acute (7 h) and chronic (7 h) DTHR (Figure 6B-C).

### Impact of NETs on the development of acute and chronic cutaneous DTHR

Recent studies have demonstrated that NETs might play a role in T cell-driven autoimmunity, acute injuries and cancer 18, 40-42. To investigate whether NET formation has an impact on the inflammatory immune response during acute and chronic cutaneous DTHR, we investigated PAD4^-/-^ mice, as it has been reported that mice deficient in PAD4 are unable to form NETs 43, 44. PAD4 is responsible for the citrullination of histones H3 and H4 by converting peptidylarginine residues into peptidylcitrullines; this process is thought to be necessary for the formation of NETs in many diseases 45, 46.

**Figure 7:**
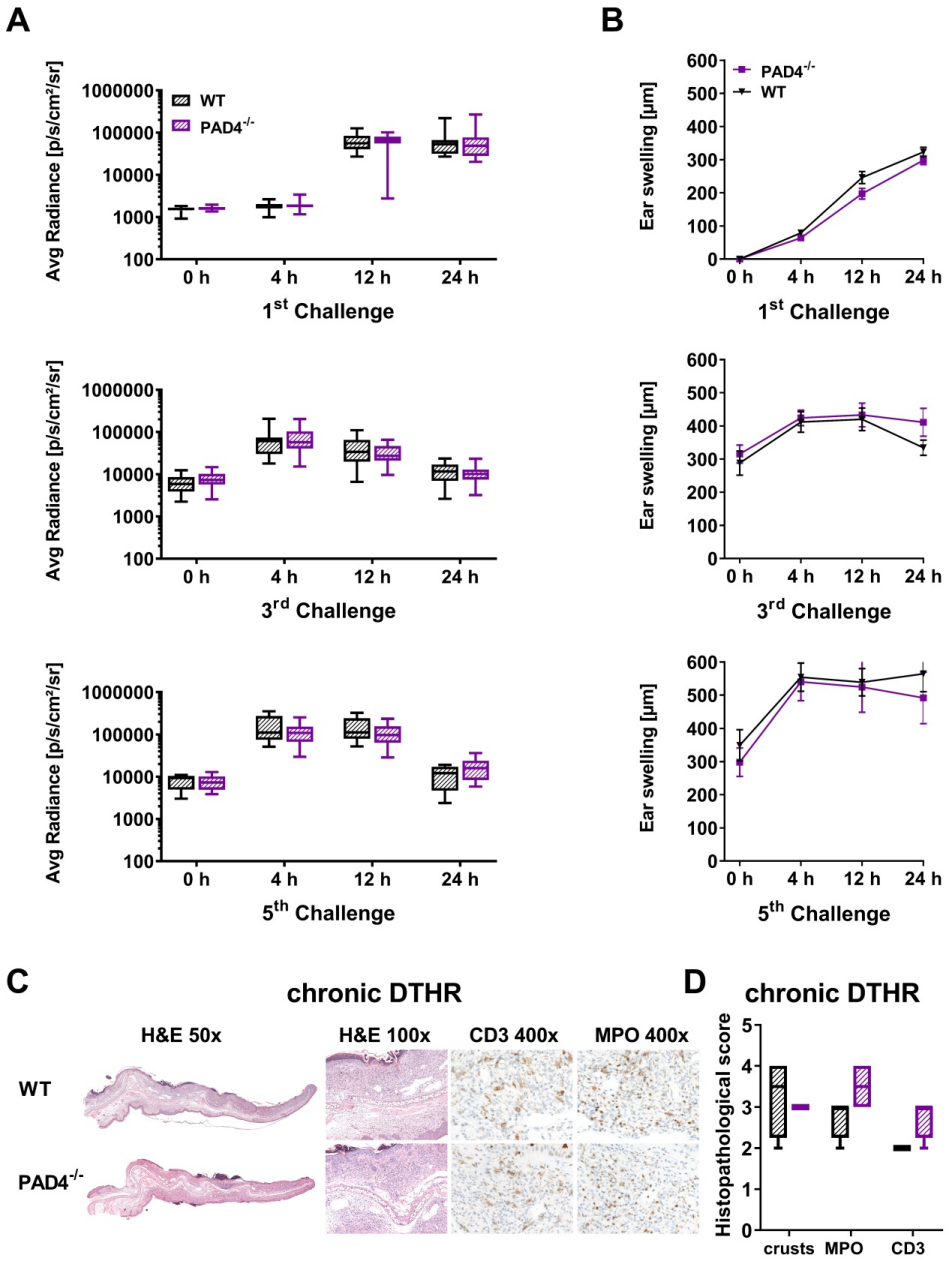
***In vivo* optical imaging of ROS/RNS production and ear swelling response in PAD4^-/-^ mice. (A)** *In vivo* optical imaging of ROS/RNS production. L-012 was injected into WT (n = 10) and PAD4^-/-^ mice (n = 10) 5 min before the optical imaging measurement. The ROS/RNS levels are displayed as the medians with interquartile ranges; whiskers indicate the min and max values of average radiance (p/s/cm^2^/sr). No significant differences in ROS/RNS production between WT and PAD4^-/-^ mice were observed (Mann-Whitney test). **(B)** Ear swelling response in PAD4^-/-^ mice in acute, early chronic and chronic DTHR after 1 % TNCB challenge. Data are displayed as the means ± SEMs. No significant difference in the ear swelling response between the PAD4^-/-^ mice and wild-type (WT) mice was observed (PAD4^-/-^: n = 10; wild-type: n = 10, unpaired, two-tailed Student's t-test). **(C)** Representative images of H&E and immunohistochemical staining of T cells (CD3) and neutrophils (MPO) in the ear tissue 24 h after the 5^th^ challenge. **(D)** Histopathological score was determined by the number of epidermal abscesses and crusts per section (0 = no crusts or abscesses; 1 = abscesses, no crusts; 2 = between 1 and 5 crusts, 3 = between 6 and 10 crusts, and 4 = more than 11 crusts). Neutrophil (MPO) abundance and T cell (CD3) abundance were determined by a semi-quantitative analysis of dermal inflammation (0 = no inflammatory infiltrate; 1 = minimal inflammatory infiltrate; 2 = mild inflammatory infiltrate; 3 = moderate inflammatory infiltrate; and 4 = severe inflammatory infiltrate). Data are displayed as the medians with interquartile ranges; whiskers indicate min and max values (n = 4).

In the inflamed ears of PAD4^-/-^ mice with acute cutaneous DTHR, compared to those in wild-type mice, we observed less NET formation and less OGG1 accumulation in the cell nuclei (Figure 6B-C). Interestingly, the nuclei of PMNs and, to some extent, the NETs stained positive for H3 citrullination (Figure S6). Thus, we determined strong NET formation in acute cutaneous DTHR that was independent of NOX2 and partially independent of PAD4 (Figure 6B-C). However, the *in vivo* L-012 OI measurements of ROS/RNS production in the inflamed ears demonstrated similar ROS/RNS values in PAD4^-/-^ and wild-type mice (Figure 7A). The time course of the ear swelling responses of PAD4^-/-^ deficient mice with cutaneous DTHR revealed no significant differences in comparison to that of wild-type mice (Figure 7B). In line with these findings, the histopathological analysis of the inflamed ears of PAD4^-/-^ mice with chronic cutaneous DTHR revealed no significant differences compared with those of wild-type mice (Figure 7C-D), indicating a negligible role of PAD4 in TNCB induced cutaneous DTHR.

## Discussion

In the present study, we investigated the selective impact of different sources of the ROS/RNS mainly produced by PMNs during acute and chronic cutaneous DTHR in mice with differently impaired ROS/RNS production.

Our *in vivo* OI results demonstrated that NOX2 and MPO are the dominant sources of ROS during acute and chronic cutaneous DTHR, while iNOS-related RNS seems to have a rather minor contribution (Figure 1A). Deficiency in NOX2, MPO and iNOS did not impair the course of the ear swelling responses during the acute, early chronic and chronic cutaneous DTHR (Figure 1B). Nevertheless, histopathological analysis revealed that NOX2 deficiency was accompanied by a rather reduced chronic DTHR, while MPO deficiency was associated with a more severe chronic DTHR (Figure 3D). However, all experimental groups revealed similar oxidative stress in the inflamed ear tissue. Furthermore, NET formation was not affected in the different mouse models of ROS/RNS deficiency and did not impact the course of acute and chronic cutaneous DTHR (Figure 6, Figure S7-8).

Our noninvasive *in vivo* OI results revealed that MPO-derived hypochlorous acid, a primary product of MPO 47, is the dominant ROS intermediate during cutaneous DTHR, since the L-012 SI was decreased by up to 90 % in the inflamed ears of MPO^-/-^ mice (Figure 1A). However, MPO relies on the presence of hydrogen peroxide, a downstream derivative of the superoxide anion. NOX2 is the primary source of superoxide anion production in PMNs and macrophages 1; several studies have also demonstrated its relevance in B and T cells 48-50. Approximately 95 % of the leucocytic infiltrate in acute cutaneous DTHR is composed of neutrophils, while in chronic cutaneous DTHR the relative numbers of neutrophils decrease and the relative numbers of T and B cells as well as other immune cells increases 51, 52. This explains the complete depletion of the L-012 SI and the low DHR FI in the ears of gp91^phox-/-^ mice (Figure 2; Suppl. Discussion 1 about L-012).

Our results suggest that iNOS plays a minor role in ROS/RNS production in acute and chronic cutaneous DTHR. A study by Abu-Soud et al. demonstrated that nitric oxide modulates the peroxidase catalytic activity of MPO by two mechanisms: 1) low levels of nitric oxide augment peroxidase activity by minimizing the accumulation of Compound II; and 2) high levels of nitric oxide promote the formation of the nitrosyl complex MPO-Fe(III)-NO, thereby reducing MPO catalysis 53. Furthermore, nitric oxide can reduce superoxide production by downregulating NOX2 54-56. Therefore, deficiency in iNOS can increase the bioavailability of superoxide for other ROS intermediates and thereby compensate for low RNS levels. Nitric oxide exhibits vaso-relaxant properties in blood vessels 57 and has inhibitory effects on platelet and PMN adhesion by downregulating the adhesion molecules intercellular adhesion molecule-1 (ICAM-1), P-selectin and α_4_β_1_-integrin, thereby playing an important role in the progression of inflammation 56, 58-61. Treatment of mice with L-N^6^-iminoethyl-lysine, an iNOS inhibitor, augmented FITC- and DNFB-induced cutaneous DTHR by enhancing the migration and survival of cutaneous DCs 62. Nevertheless, Ross et al. previously showed that acute cutaneous DTHR was significantly reduced by the administration of aminoguanidine, which preferentially inhibits the iNOS isozyme 63. These data support our histopathological findings in the inflamed ears of iNOS^-/-^ mice, even though deficiency in iNOS had no impact on ear swelling responses and PMN recruitment (Figure 3). Similar results were shown by others, where inhibition of nitric oxide by aminoguanidine increased PMN migration in experimental sepsis, while iNOS-deficient mice did not display differences in PMN migration compared to that in wild-type mice 64, 65.

Furthermore, our results revealed that MPO deficiency-related impairment of ROS production is associated with increased chronic inflammation, indicating an anti-inflammatory role of MPO in chronic cutaneous DTHR. MPO is a key mediator of tissue damage and organ inflammation in many inflammatory diseases that acts through the generation of reactive oxidizing agents 66-68. Several studies have demonstrated that MPO deficiency augments autoimmune diseases, such as pulmonary inflammation, atherosclerosis, skin inflammation, rheumatoid arthritis and multiple sclerosis, by affecting adaptive immunity 69, 70. Odobasic et al. showed that MPO^-/-^ mice developed increased ovalbumin-induced cutaneous DTHR and antigen-induced arthritis, which were associated with enhanced T cell activation and proliferation due to increased DC activation, antigen processing and migration to the lymph nodes 71. Similar results were observed in an experimental model of lupus nephritis, where MPO deficiency led to increased renal injury with enhanced DC activation and migration to the secondary lymphatic organs and increased accumulation of PMNs, CD4^+^ T cells and macrophages in the glomeruli 72. However, the exact mechanism of how MPO affects DCs is still to be identified. Various reports lead to the assumption that MPO-derived HOCl might induce conformational changes in Mac-1, an inhibitory receptor of DCs, thereby inhibiting DC function and therefore might interfere with the subsequent adaptive immune response 70. Although inflamed ears of MPO^-/-^ mice displayed increased PMN and T cell accumulation (Figure 3D), we observed no differences in the relative number and activation states of T cells or in the accumulation of DCs in the dLNs or spleens of experimental wild-type, gp91^phox-/-^, MPO^-/-^ and iNOS^-/-^ mice (Figure S4). This suggests that the proinflammatory effects of MPO deficiency in chronic DTHR are mainly due to the altered neutrophil function 69. Several *in vitro* and *in vivo* studies have demonstrated the upregulation proinflammatory cytokines and chemokines in MPO^-/-^ PMNs after stimulation with zymosan, an antagonist for TLR-2 similar to TNCB 73-75. Tateno et al. demonstrated that exogenous H_2_O_2_ treatment in addition to zymosan enhanced the phosphorylation of ERK1/2. In contrast, exogenous treatment with HOCl inhibited the NF-κB activity, suggesting that both lack of HOCl and accumulation of H_2_O_2_ due to MPO deficiency contribute to the upregulation of proinflammatory cytokines in the neutrophils 75. Further, HOCl can inhibit the expression of endothelial adhesion molecules or disrupt the adhesive properties of subendothelial matrix 76-78. Therefore, deficiency in MPO could contribute to enhanced neutrophil infiltration in the inflamed ear tissue observed in our experiments.

An analysis of lipid peroxidation in the cell homogenates of inflamed ears revealed slightly enhanced values in the inflamed ears of MPO^-/-^ mice but slightly decreased values in the inflamed ears of iNOS^-/-^ mice in comparison to wild-type mice (Figure 5B). Brovkovych et al. reported that MPO deficiency increases nitric oxide and RNS intermediates by enhancing the expression and activity of iNOS 79, which could contribute to the lipid peroxidation observed in our experiments. However, we couldn't detect any upregulation of iNOS mRNA expression in the inflamed ear of MPO^-/-^ mice (data not shown). In addition, comparison of MPO^-/-^ mice with wild-type mice, undergoing acute lipopolysaccharide-induced lung inflammation, revealed similar expression of HO-1, an indicator of oxidative stress 80, which is congruent to our results. Nevertheless, MPO deficiency is beneficial in various inflammatory diseases, such as cardiovascular disease, neuronal disease, pulmonary and renal inflammation 69. This finding suggests that the balance between MPO-induced local tissue injury and MPO-associated downregulation of adaptive immunity is unique for each inflammatory condition, thereby determining the outcome of the disease 70.

Our investigations revealed opposing results on the impact of ROS/RNS on chronic cutaneous DTHR in MPO^-/-^ and gp91^phox-/-^ mice, as we observed increased inflammation in MPO^-/-^ mice and, in contrast, decreased inflammation in gp91^phox-/-^ mice (Figure 3D). Although the expression of *Tnf* mRNA was significantly enhanced in inflamed ears of gp91^phox-/-^ mice with chronic cutaneous DTHR (Figure 4A), these mice failed to exhibit a strong inflammatory phenotype.

Several studies have demonstrated that ROS can regulate both the apoptotic signaling and NF-κB transcription triggered by TNF 81-83. Thus, ROS can mediate TNF-induced apoptosis through the sustained activation of JNK or by the inhibition of the NF-κB survival pathway, thereby promoting inflammation and injury of the tissue 84-87. Therefore, impaired ROS/RNS production in the ears of gp91^phox-/-^ mice could dampen the effect of TNF and contribute to reduced inflammation. Nevertheless, NOX2 deficiency did not protect the animals from oxidative tissue damage (Figure 5A-B, E). A study by Sundqvist et al. demonstrated that patients with chronic granulomatous disease lacking functional NOX2, displayed elevated mitochondrial ROS production and oxidative stress 88. However, additional scavenging of mitochondrial ROS in NOX2-deficient mice in our experiments had no effect on the course of the acute and chronic cutaneous DTHR (Figure S6). This suggests that other sources of ROS probably contributed to oxidative stress. Indeed, all experimental groups exhibited an increased expression of xanthine oxidase in acute and chronic DTHR (Figure 4C). In an experimental model of LPS-induced skin inflammation that is independent of an adaptive immune response, Nakai et al. demonstrated that not NOX2, but xanthine oxidase and iNOS, are the main sources of ROS/RNS 89. Other NOX isoforms could also contribute to oxidative stress. *In vitro* studies have demonstrated that NOX4 is the main ROS source in human psoriatic fibroblasts and is essential for redox-mediated modulation of the metabolism in keratinocytes 90, while inhibition of NOX1 in keratinocytes derived from the skin of atopic dermatitis patients significantly reduced ROS production 91. Furthermore, NOX4 is highly expressed in endothelial cells and is involved in osteoarthritis, renal diseases and angiogenesis during ischemia, hypoxia and inflammation 92-94. Therefore, resident cells like keratinocytes, fibroblasts and endothelial cells could also contribute to local ROS/RNS production and oxidative stress through other NOX isoforms in the inflamed ears of gp91^phox-/-^ mice. However, whether these *in vitro* findings are fully transferable to living organisms requires further investigation since the interaction between different resident and infiltrating immune cells might affect their metabolism and function substantially.

Remarkably, NOX2 deficiency is associated with autoimmunity and immune-mediated inflammatory diseases in humans and rodents 95-97. For example, mice with a defect in Ncf1, a cytosolic subunit of NOX2, develop severe collagen-induced arthritis and EAE due to the enhanced activation of autoreactive T cells 98. One mechanism by which NOX2 can regulate the immune response is the modification of surface redox levels of T cells 99. Thus, ROS can directly regulate T cells by oxidizing the T cell receptor zeta or cofilin, resulting in T cell hypo-responsiveness 100, 101. In contrast, NOX2-derived ROS intermediates can also cause antigen leakage from endosomes through increased lipid peroxidation and cell membrane disruption, thereby increasing cross-presentation via MHC class I to CD8^+^ T cells 102. Although lipid peroxidation in the inflamed ears of gp91^phox-/-^ mice was slightly reduced compared to that in wild-type mice, our flow cytometry analysis revealed no differences in T cell activation and differentiation (Figure S4), suggesting that they were not affected by reduced ROS/RNS production in gp91^phox-/-^ mice.

Our metabolomic analysis revealed elevated GSSH levels in all investigated genotypes with chronic DTHR, indicating similar oxidative stress in all experimental groups (Figure 5C-E). Under normal physiological conditions, 98 % of glutathione (GSH) is present in its reduced form 103. Importantly, during oxidative stress, GSH is oxidized to GSSH and its recovery back to reduced GSH is strongly dependent on NADPH (reduced form of NAD^+^) 104. The NAD^+^ glycohydrolase (CD38) is a major NAD^+^ consuming enzyme and its expression is highly elevated in a variety of cells during inflammation 105, 106. This could explain the reduced levels of NAD^+^ in inflamed ears with chronic DTHR. Furthermore, low levels of NAD^+^ can contribute to increased GSSH levels and reinforce the oxidative stress measured in our experiments (Figure 5). Also depletion of arginine and its precursor proline 107 which are necessary for the biosynthesis of nitric oxide (NO) 108 suggest a strong activity of nitric oxide synthases in chronic cutaneous DTHR. Interestingly, inflamed ears of mice with chronic DTHR exhibited strongly elevated ascorbate (vitamin C) levels in comparison to healthy ears of naïve mice (Figure 5E). Ascorbate is an important antioxidant and a radical scavenger. Consequently, mice which are unable to synthesize ascorbate display elevated oxidative stress levels 109. In line with this, our metabolic analysis results reveal an upregulation of ascorbate synthesis due to inflammation related enhanced oxidative stress. Nevertheless, the exact impact of inflammation on ascorbate synthesis needs to be further investigated.

NOX2 or mitochondria-related ROS are also heavily involved in NET formation 110. NETs are chromatin structures decorated with different PMN granule proteins, such as MPO, histones and neutrophil elastase released by PMNs into the extracellular space after exposure to pathogens or inflammatory stimuli 96, 110. Currently, two main mechanisms have been described: NOX-dependent and NOX-independent NET formation 110. Since NETs were present in gp91^phox-/-^ mice (Figure 6A-B), our results suggest that NET formation in TNCB-induced cutaneous DTHR is NOX2-independent. Furthermore, NETs in the inflamed ears of wild-type mice were highly positive for H3 citrullination, but not in inflamed ears of gp91^phox-/-^ mice (Figure S7), indicating that NOX2 is an upstream regulator of H3 citrullination. In contrast, previous studies proposed that PAD4-mediated H3 citrullination is required for NOX2-independent NET formation but not for NOX2-dependent NET formation 110-113. However, the assumption that PAD4-mediated histone citrullination is required for NET formation has never been proven, and recent studies have demonstrated PAD4-independent NET formation 110, 112, 114. Citrullination of histone H3 can also be promoted by PAD2 115, 116, which is highly expressed in PMNs and could explain H3 citrullination in PAD4^-/-^ mice. A recent study on human PMNs by Zhou et al. demonstrated that PAD4-mediated citrullination is directly linked to the oxidative burst 117. PAD4, in its inactive form, is physically linked to the cytosolic subunits p47^phox^ and p67^phox^ of NADPH oxidase. The activation of PAD4 causes the citrullination of p47^phox^ and p67^phox^, followed by their dissociation from PAD4, thereby preventing active NADPH oxidase assembly and consequently ROS production 117. However, our *in vivo* OI results revealed no difference in ROS/RNS production in PAD4^-/-^ mice when compared to that in wild-type mice (Figure 7A). This discrepancy could be due to species-related differences between mouse and human PMNs 118, 119. For example, after challenge with Aspergillus fumigatus, phosphatidylinositol 3-phosphate binding to p40^phox^ is necessary for NADPH oxidase activation and oxidative burst in human, but not murine PMNs 120. Furthermore, Lood et al. reported that the mitochondrial and chromosomal DNA in NETs contains high amounts of 8-OHdG 121, which could explain the pronounced accumulation of OGG1 within NETs, qualifying OGG1 staining as an ideal marker for the identification of NETs in tissues.

It is also important to determine whether oxidative stress is the primary cause of disease or a kind of bystander product during disease progression 21. Thus, antioxidant therapies may be ineffective in diseases in which oxidative stress is not the initiating factor and main cause of the disease 21 (Suppl. Discussion 2 about antioxidant treatment approaches). This could also be the reason for the marginal effect of ROS/RNS impairment on acute and chronic cutaneous DTHR in our experiments, since ROS/RNS production seems to be rather a bystander product “surrogate marker” and not the initial cause. Generally, redox signaling is a very complex system in which numerous pathways and ROS/RNS sources interact with each other, and deficiency in one source of ROS/RNS might be compensated by enhanced expression or activity of other sources of ROS/RNS 53, 79.

## Conclusions

NOX2 seems to play an anti-inflammatory role in acute DTHR but a proinflammatory role in chronic DTHR. In contrast, MPO deficiency might promote chronic cutaneous DTHR. Additionally, acute but not chronic DTHR was accompanied by nuclear NET formation in a NOX2-independent manner. Nevertheless, deficiency in the predominant sources of ROS/RNS in MPO^-/-^, gp91^phox-/-^, iNOS^-/-^ mice exhibited only marginal differences in the progression of disease and did not protect the mice from oxidative stress indicating a minor role of phagocyte-derived ROS/RNS in the inflammatory process during acute and chronic DTHR. Consequently, deficiency in one dominant source of ROS/RNS might be successfully compensated by other sources of ROS/RNS. Moreover, moderate non-phagocytic ROS/RNS production seems to be sufficient for modulation of acute and chronic cutaneous DTHR. Therefore, proper therapeutic targeting of more than one dominant source of ROS/RNS might be essential for an efficient treatment approach.

## Supplementary Material

Supplementary discussion, figures, table.

## Abbreviations

8-OHdG: 8-hydroxy-2'-deoxyguanosine
ANOVA: analysis of variance
Ccl2: chemokine (C-C motif) ligand 2
Cxcl1/2: chemokine (C-X-C motif) ligand 1/2
DCs: dendritic cells
DHR: dihydrorhodamin 123
dLN: draining lymph node
DMF: dimethyl fumarate
DTHR: delayed-type hypersensitivity reaction
eNOS: endothelial nitric oxide synthase
FI: fluorescence intensity
Gpx1: glutathione peroxidase
GSH: glutathione
GSSG: glutathione disulfide
HCA: hierarchical cluster analysis
H&E: hematoxylin and eosin
Hmox1: heme oxygenase 1
HOCl: hypochlorous acid
Il1-beta: interleukin 1 beta
Il6: interleukin 6
iNOS: inducible nitric oxide synthase
MDA: malondialdehyde
MFI: mean fluorescence intensity
MPO: myeloperoxidase
NAD: nicotinamide adenine dinucleotide
NETs: neutrophil extracellular traps
NF-κB: nuclear factor kappa-light-chain-enhancer of activated B cells
NOX2: NADPH oxidase 2 (gp91^phox^)
Nrf2: nuclear factor erythroid 2-related factor 2
Ogg1: 8-oxoguanine glycosylase1
OI: optical imgaging
PAD4: peptidylarginine deiminase 4
PC: principal component
PCA: principal component analysis
PMA: phorbol-12-myristat-13-acetate
PMNs: polymorphonuclear neutrophils
PQN: percentile quantile normalization
RNS: reactive nitrogen species
ROS: reactive oxygen species
SEM: standard error of the mean
SI: signal intensity
Sod1: superoxide dismutase 1
TNCB: 2,4,6-trinitrochlorobenzene
TNF: tumor necrosis factor
Xhd: xanthine oxidase
