# Supplementary material for: Immunomodulatory role of reactive oxygen species and nitrogen species during T cell-driven neutrophil-enriched acute and chronic cutaneous delayed-type hypersensitivity reactions
Source: Theranostics. 2021 Jan 1;11(2):470–90. doi: 10.7150/thno.51462 (PMC7738859; doi:10.7150/thno.51462)
Supplement: Supplementary file 1 — Supplementary discussion, figures, table. [file thnov11p0470s1.pdf]

Immunomodulatory role of reactive oxygen species and nitrogen species during T cell-driven neutrophil-enriched acute and chronic cutaneous delayed-type hypersensitivity reactions

Roman Mehling[1], Johannes Schwenck[1,2,3], Christina Lemberg[4], Christoph Trautwein[1], Laimdota Zizmare[1], Daniela Kramer[5], Anne Müller[5], Birgit Fehrenbacher[6], Irene Gonzalez-Menendez[7], Leticia Quintanilla-Martinez[7], Katrin Schröder[8], Ralph P. Brandes[8], Martin Schaller[6], Wolfram Ruf[9], Martin Eichner[10], Kamran Ghoreschi[11], Martin Röcken[3,6], Bernd J. Pichler[1,3], Manfred Kneilling[1,3,6]*

* Corresponding author email: manfred.kneilling@med.uni-tuebingen.de
telephone: +49-7071-29-86870
fax: +49-7071-29-4451

[1]Werner Siemens Imaging Center, Department of Preclinical Imaging and Radiopharmacy, Eberhard Karls University, Tübingen, Germany.
[2]Department of Nuclear Medicine and Clinical Molecular Imaging, Eberhard Karls University, Tübingen, Germany.
[3]Cluster of Excellence iFIT (EXC 2180) "Image-Guided and Functionally Instructed Tumor Therapies", Eberhard Karls University, 72076 Tübingen, Germany
[4]Section of Immunology, Vetsuisse Faculty, University of Zürich, Zürich, Switzerland.
[5]Interfaculty Institute for Biochemistry, Eberhard Karls University, Tübingen, Germany.
[6]Department of Dermatology, Eberhard Karls University, Tübingen, Germany.
[7]Department of Pathology, Eberhard Karls University, Tübingen, Germany
[8]Institute for Cardiovascular Physiology, Goethe-University, Frankfurt, Germany.
[9]Center for Thrombosis and Hemostasis, University Medical Center Mainz, Mainz, Germany.
[10]Institute for Clinical Epidemiology and Applied Biometry, Eberhard Karls University, Tübingen, Germany
[11]Department of Dermatology, Venereology and Allergology, Charité - Universitätsmedizin Berlin, Berlin, Germany.

Supplementary Discussion 1

L-012 is ideal for imaging ROS and RNS in PMNs, since it has a very high sensitivity towards hypochlorous acid, hydroxyl radical and peroxynitrite, which are produced at high concentration during the oxidative burst and can directly oxidize the chemiluminescent probe [1-4]. Although previously assumed to be highly sensitive towards superoxide [1, 5], L-012 does not directly react with superoxide, as it first needs to be oxidized by hydrogen peroxide in the presence of peroxidases or other ROS/RNS intermediates [6]. While DHR is also known to react well with hydrogen peroxide in the presence of peroxidases in addition to the abovementioned ROS/RNS intermediates [7-9], L-012 is not a very efficient substrate for direct reaction with peroxidases, since a high concentration of L-012 is needed [6]. This inefficient reaction could be the reason for the higher ROS/RNS levels observed in the ears of $MPO^{-/-}$ mice by *ex vivo* DHR flow cytometry in comparison to wild-type mice (Figure 2) than the difference observed by *in vivo* L-012 OI measurements (Figure 1A). Since MPO is the most abundant protein in PMNs [10], MPO deficiency could mask the real amount of hydrogen peroxide or superoxide measured by L-012. However, the comparison of *in vivo* with *ex vivo* measurements is always difficult, since leukocytes derived from the inflamed ears are remarkably affected by the homogenization of the ear tissue, which does not apply to *in vivo* measurements.

Supplementary Discussion 2

Multiple studies have demonstrated the contribution of oxidative stress to the pathogenesis of different diseases, especially in neutrophil-enriched autoimmune diseases such as rheumatoid arthritis, psoriasis vulgaris, ulcerative colitis and Crohn’s disease [11-13]. Unfortunately, most antioxidant treatment approaches failed in clinical trials, as they yielded no or only limited beneficial effects [14, 15], which has created a pessimistic mindset towards antioxidant therapies. The reasons for the failure of antioxidant treatments are diverse, e.g., low bioavailability or dosage, inappropriate administration time point, frequency and duration of the therapy, poor specificity or harmful side effects that mask the beneficial antioxidant action [15]. In addition, some of the antioxidative compounds that are successfully used in daily clinical practice are not antioxidants per se, as they exhibit multiple off-target effects. For example, dimethyl fumarate (DMF) is approved for the treatment of multiple sclerosis [16] and psoriasis [17]. It is assumed that DMF activates the Nrf2 pathway, which regulates the expression of various antioxidant proteins and restores the redox balance [18, 19]. However, DMF is observed to modulate the innate and adaptive immune systems in Nrf2-deficient mice [20] and to inhibit the expression of inflammatory cytokines and adhesion molecules by inhibiting the translocation of NF-κB [19, 21]. These diverse observations make it difficult to conclude whether the therapeutic benefit of DMF is due to antioxidative or immunomodulatory effects.

Supplementary References

1. Daiber A, Oelze M, August M, Wendt M, Sydow K, Wieboldt H, et al. Detection of superoxide and peroxynitrite in model systems and mitochondria by the luminol analogue L-012. Free Radic Res. 2004; 38: 259-69.
2. Imada I, Sato EF, Miyamoto M, Ichimori Y, Minamiyama Y, Konaka R, et al. Analysis of reactive oxygen species generated by neutrophils using a chemiluminescence probe L-012. Anal Biochem. 1999; 271: 53-8.
3. Kielland A, Blom T, Nandakumar KS, Holmdahl R, Blomhoff R, Carlsen H. In vivo imaging of reactive oxygen and nitrogen species in inflammation using the luminescent probe L-012. Free Radic Biol Med. 2009; 47: 760-6.
4. Zhou J, Tsai YT, Weng H, Tang L. Noninvasive assessment of localized inflammatory responses. Free Radic Biol Med. 2012; 52: 218-26.
5. Daiber A, August M, Baldus S, Wendt M, Oelze M, Sydow K, et al. Measurement of NAD(P)H oxidase-derived superoxide with the luminol analogue L-012. Free Radic Biol Med. 2004; 36: 101-11.
6. Zielonka J, Lambeth JD, Kalyanaraman B. On the use of L-012, a luminol-based chemiluminescent probe, for detecting superoxide and identifying inhibitors of NADPH oxidase: a reevaluation. Free Radic Biol Med. 2013; 65: 1310-4.
7. Crow JP. Dichlorodihydrofluorescein and dihydrorhodamine 123 are sensitive indicators of peroxynitrite in vitro: implications for intracellular measurement of reactive nitrogen and oxygen species. Nitric Oxide. 1997; 1: 145-57.
8. Royall JA, Ischiropoulos H. Evaluation of 2',7'-dichlorofluorescin and dihydrorhodamine 123 as fluorescent probes for intracellular H2O2 in cultured endothelial cells. Arch Biochem Biophys. 1993; 302: 348-55.
9. Dypbukt JM, Bishop C, Brooks WM, Thong B, Eriksson H, Kettle AJ. A sensitive and selective assay for chloramine production by myeloperoxidase. Free Radic Biol Med. 2005; 39: 1468-77.
10. Klebanoff SJ, Kettle AJ, Rosen H, Winterbourn CC, Nauseef WM. Myeloperoxidase: a front-line defender against phagocytosed microorganisms. J Leukoc Biol. 2013; 93: 185-98.
11. Tian T, Wang Z, Zhang J. Pathomechanisms of Oxidative Stress in Inflammatory Bowel Disease and Potential Antioxidant Therapies. Oxid Med Cell Longev. 2017; 2017: 4535194.
12. Lin X, Huang T. Oxidative stress in psoriasis and potential therapeutic use of antioxidants. Free Radic Res. 2016; 50: 585-95.
13. Quinonez-Flores CM, Gonzalez-Chavez SA, Del Rio Najera D, Pacheco-Tena C. Oxidative Stress Relevance in the Pathogenesis of the Rheumatoid Arthritis: A Systematic Review. Biomed Res Int. 2016; 2016: 6097417.
14. Iannitti T, Palmieri B. Antioxidant therapy effectiveness: an up to date. Eur Rev Med Pharmacol Sci. 2009; 13: 245-78.
15. Firuzi O, Miri R, Tavakkoli M, Saso L. Antioxidant therapy: current status and future prospects. Curr Med Chem. 2011; 18: 3871-88.
16. Tecfidera (dimethyl fumarate). In: FDA, editor.; 2013.
17. Skilarance (dimethyl fumarate), EMEA/H/C/002157. https://wwwemaeuropaeu/en/medicines/human/EPAR/skilarence: European Medicines Agency; 2017.
18. Linker RA, Gold R. Dimethyl fumarate for treatment of multiple sclerosis: mechanism of action, effectiveness, and side effects. Curr Neurol Neurosci Rep. 2013; 13: 394.

19. Bruck J, Dringen R, Amasuno A, Pau-Charles I, Ghoreschi K. A review of the mechanisms of action of dimethylfumarate in the treatment of psoriasis. Exp Dermatol. 2018; 27: 611-24.
20. Schulze-Topphoff U, Varrin-Doyer M, Pekarek K, Spencer CM, Shetty A, Sagan SA, et al. Dimethyl fumarate treatment induces adaptive and innate immune modulation independent of Nrf2. Proc Natl Acad Sci U S A. 2016; 113: 4777-82.
21. Mrowietz U, Asadullah K. Dimethylfumarate for psoriasis: more than a dietary curiosity. Trends Mol Med. 2005; 11: 43-8.

Table S1

gene	forward primer	reverse primer
Actin	AGGAGTACGATGAGTCCGGC	GGTGTAAAACGCAGCTCAGTA
Tnf	AAGTTCCCAAATGGCCTCCC	TTGCTACGACGTGGGCTAC
Il1b	AGCTGAAAGCTCTCCACCTC	GCTTGGGATCCACACTCTCC
Il6	GTCCGGAGAGGAGACTTCAC	GCAAGTGCATCATCGTTGTTC
Ccl2	CTGGAGCATCCACGTGTTGG	CCCATTCCTTCTTGGGGTCAG
Cxcl1	ACGTGTTGACGCTTCCCTTG	TCCTTTGAACGTCTCTGTCCC
Cxcl2	CGCCCAGACAGAAGTCATAGC	CTTTGGTTCTTCCGTTGAGGG
Nrf2	TAGTTCTCCGCTGCTCGGAC	TGTCTTGCCTCCAAAGGATGTC
Hmox1	TGACACCTGAGGTCAAGCAC	AAGTGACGCCATCTGTGAGG
Gpx1	GTTCGGACACCAGGAGAATGG	TAAAGAGCGGGTGAGCCTTC
Sod1	ACTTCGAGCAGAAGGCAAGC	CCAGGTCTCCAACATGCCTC
Ogg1	AGCTTCTGGACAGTCCTTCCG	AGTACTTGTGTAGGGTTTCCAGC
Xhd	TGACGAGGACAACGGTAGATG	TCTGAAGGCGGTCATACTTGG

Figure S1

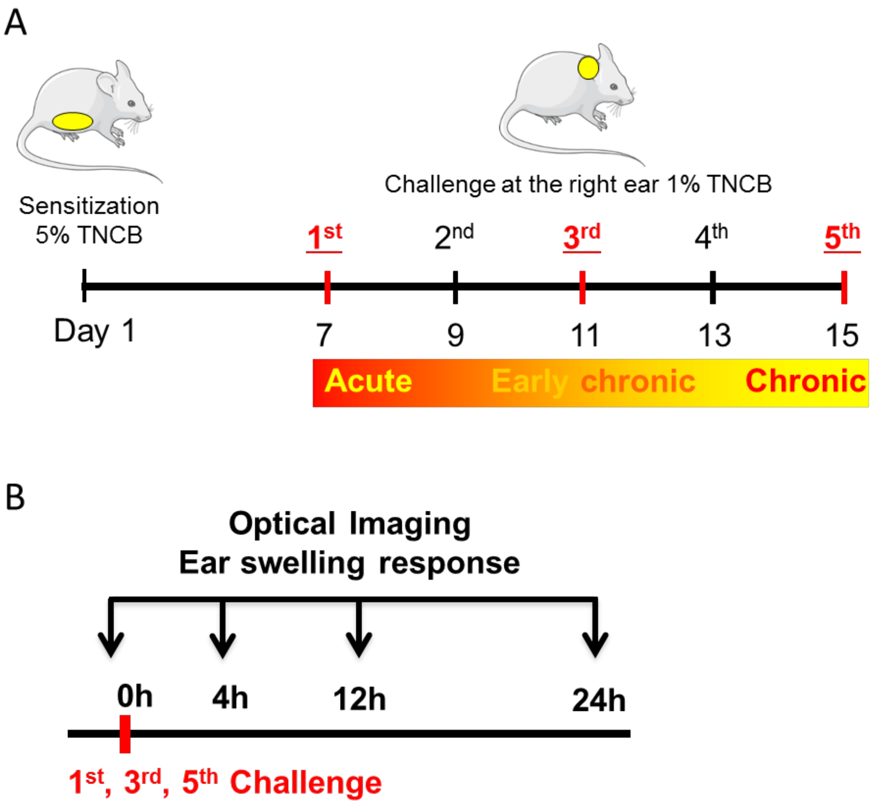

Experimental setup. A: Scheme of acute, early chronic and chronic cutaneous DTHR. **B**: Investigated time points for optical imaging (L-012) and ear swelling responses.

Figure S2

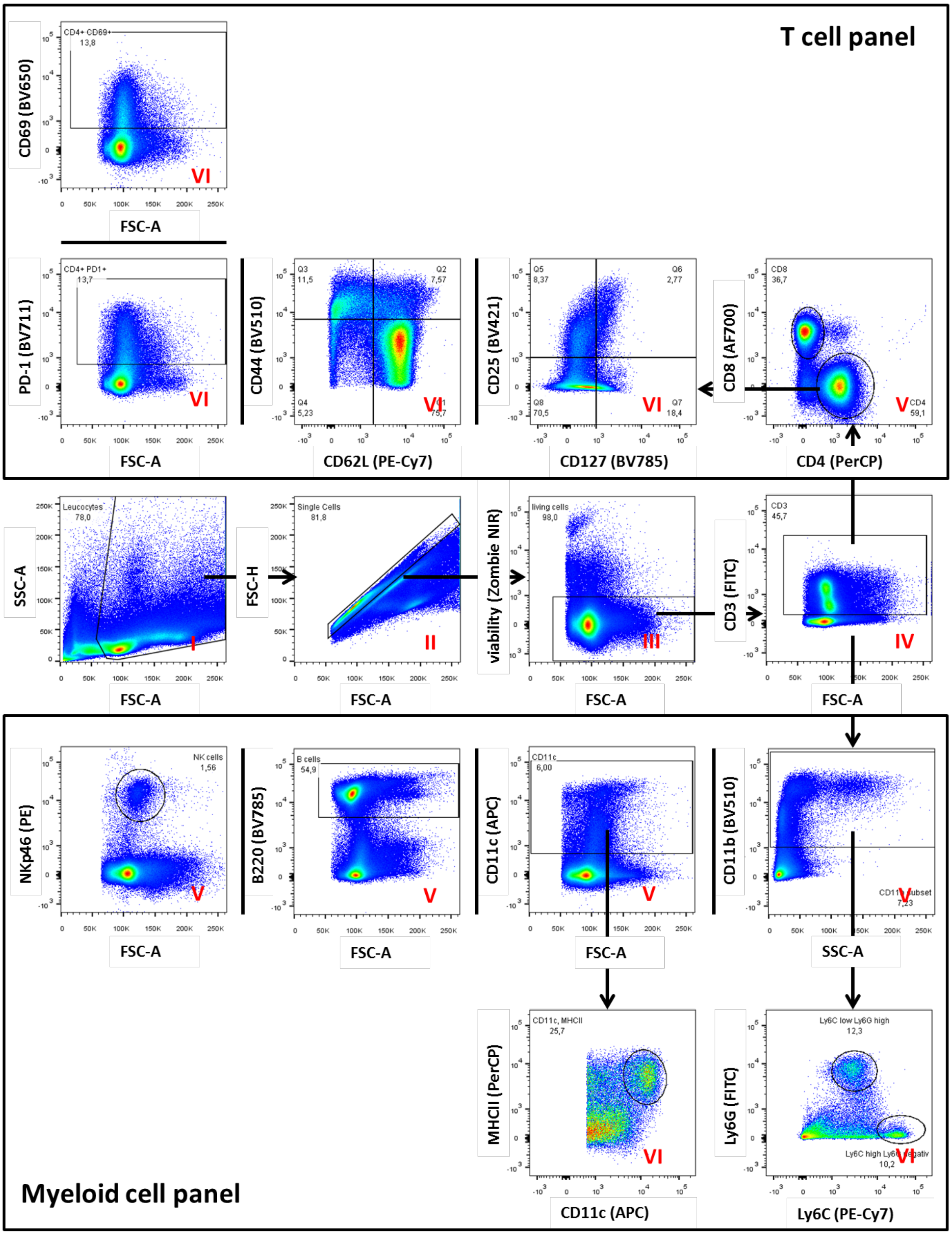

Flow cytometry gating strategy. The cells were gated based on SSC and FSC (I) and then gated for single cells (II) followed by Zombie^{neg} gating for viable cells (III). For the T cell panel, the cells were gated for CD3^{+} (IV) followed by CD4^{+} and CD8^{+} gating (V at top) for T cells. Memory T cells were defined as CD62L^{low} + CD44^{high}, naïve T

cells were defined as $CD62L^{high}$ and $CD44^{low}$, and regulatory T cells were defined as $CD127^{low}$ and $CD25^{high}$. For the myeloid cell panel, the cells were gated for $CD3^{-}$ (IV). The cells were defined as follows: NK cells as $NKp46^{+}$, B cells as $B220^{+}$, DCs as $CD11c^{+}$ and $MHCII^{+}$, monocytes as $CD11b^{+}/Ly6G^{-}/Ly6C^{+}$, and neutrophils as $CD11b^{+}/Ly6G^{+}/Ly6C^{low}$.

Figure S3

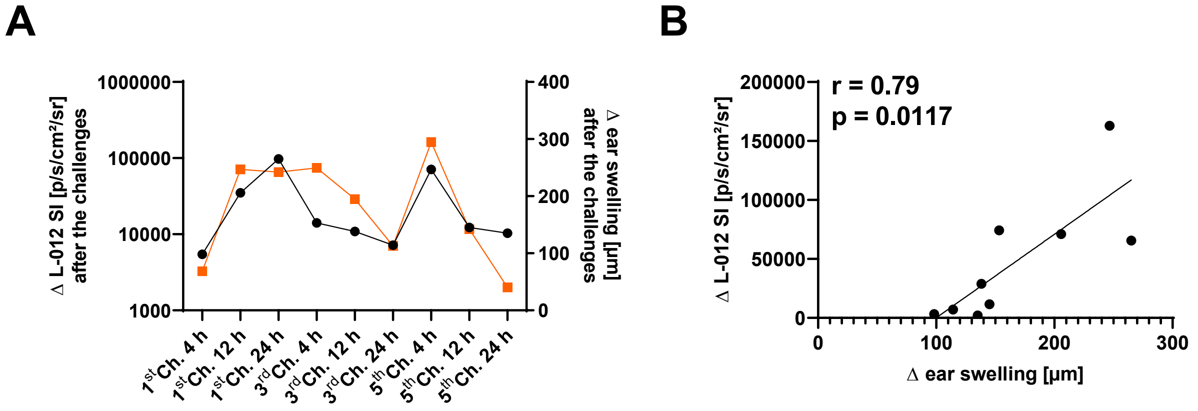

A: Differences (delta (Δ)) in ear swelling and L-012 SI between the baseline before TNCB challenge and the indicated timepoints after the challenges in wild-type mice. B: Correlation between Δ ear swelling and delta L-012 SI in wild-type mice. Pearson correlation coefficient r = 0.79, p = 0.0117 (Two-tailed).

Figure S4

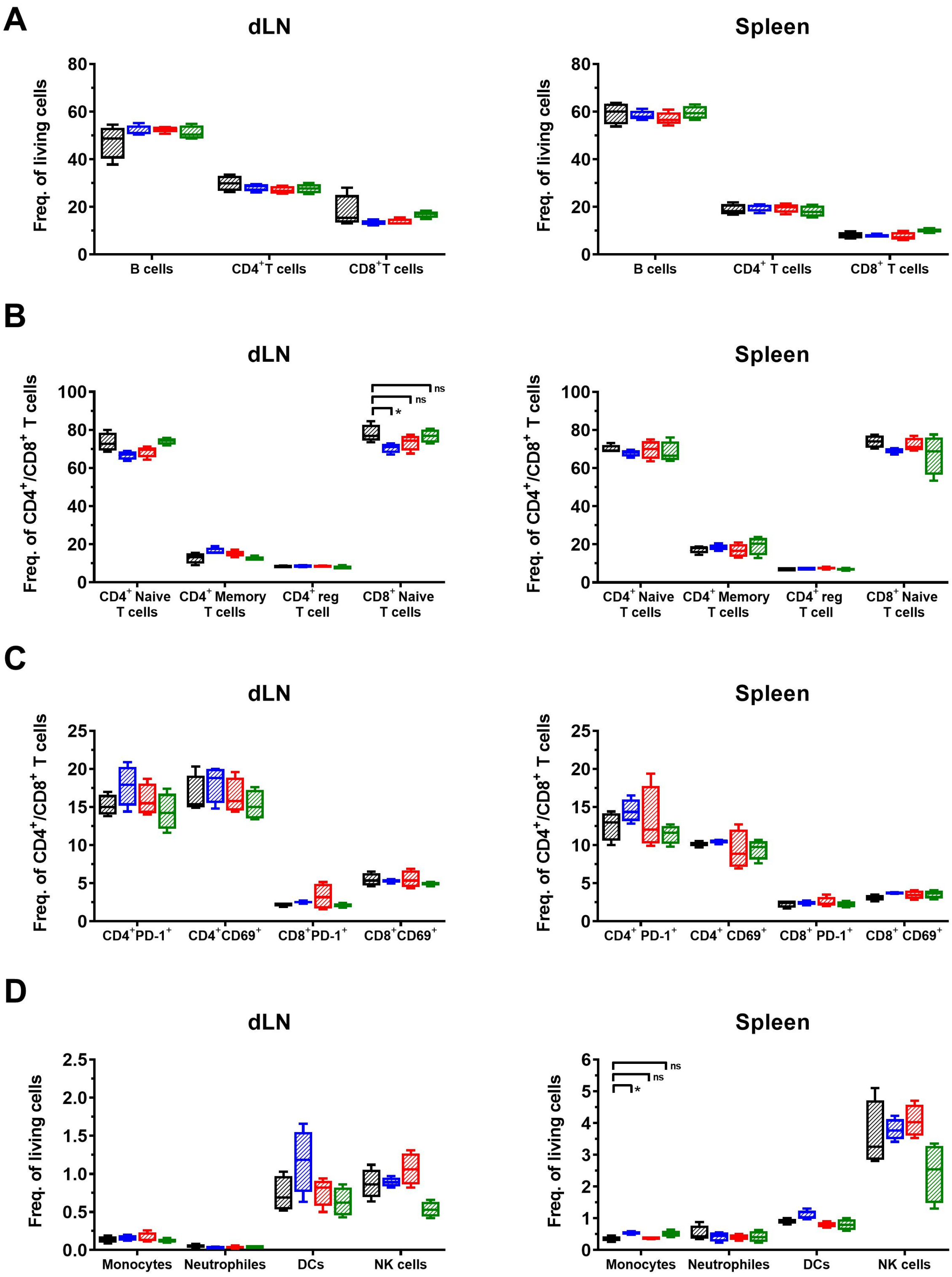

Flow cytometry analysis of the cell populations in the draining lymph nodes (dLN) and spleens in chronic DTHR (24 h after the 5th 1% TNCB challenge). A: Frequency of B and T cells. B: Composition of T cells. C: Expression of T cell activation marker CD69 and checkpoint PD-1 on CD4 and CD8 positive T cells. D: Composition of the

leukocyte population. Data are expressed as the medians with interquartile ranges; whiskers indicate the min and max values; *$p < 0.05$, ns = not significant (Kruskal-Wallis tests with post hoc Dunn tests).

Figure S5

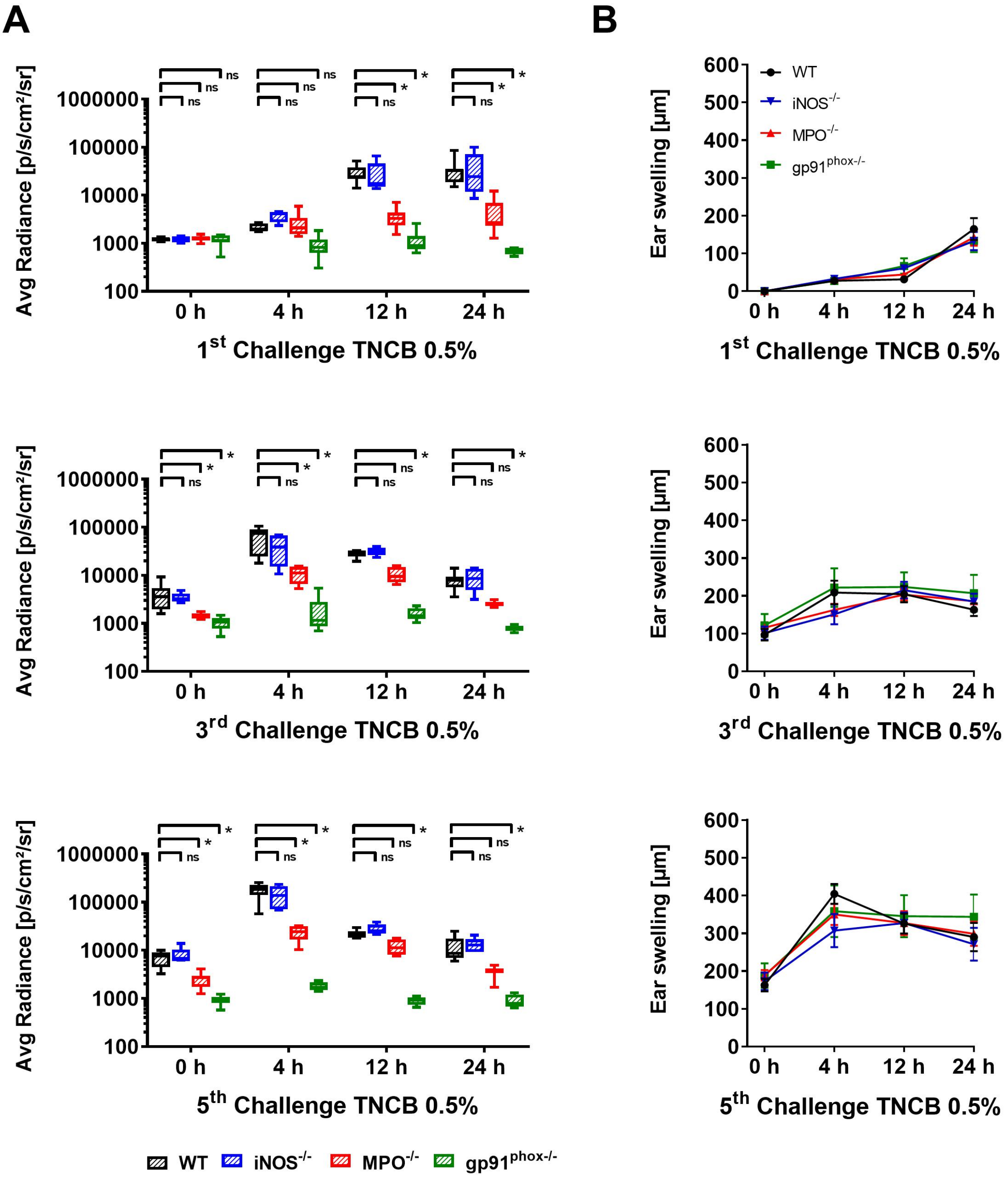

ROS/RNS production (A) and ear swelling response (B) after the 1st, 3rd and 5th 0.5% TNCB challenge. Ear swelling responses are displayed as the mean ± SEM. ROS/RNS production is displayed as the medians with interquartile ranges; whiskers indicate the min and max values; *p < 0.05, ns = not significant (Kruskal-Wallis tests with post hoc Dunn tests), WT (n=7), iNOS$^{-/-}$ (n=5), MPO$^{-/-}$ (n=7) and gp91$^{phox-/-}$ (n=6) mice.

Figure S6

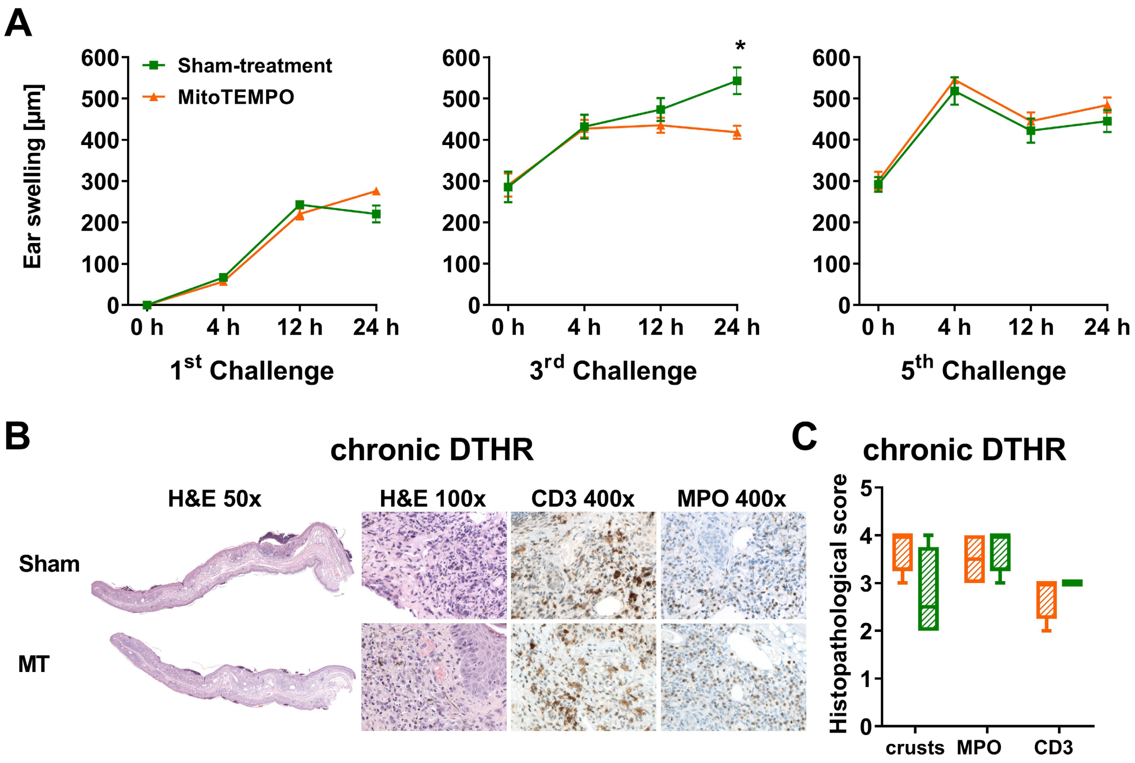

Inhibition of mitochondrial ROS production in gp91$^{phox-/-}$ mice. **(A)** Ear swelling response in gp91$^{phox-/-}$ mice to repetitive challenge with a 1% TNCB solution after treatment with MitoTEMPO (MT) or Sham (NaCl). MitoTEMPO 1.5 mg/kg or a Sham treatment was administered *i.p.* daily, starting three days before the first TNCB challenge. Data are displayed as the means ± SEMs. The only significant difference in ear swelling response between the MitoTEMPO and Sham treatment groups was observed 24 h after the 3rd challenge (treatment group: n=9; control group: n=8, unpaired, two-tailed Student's t-test). **(B)** Representative images of H&E and immunohistochemical staining of T cells (CD3) and neutrophils (MPO) in ear tissue 24 h after the 5th challenge. **(C)** The histopathological score was determined by number of epidermal abscesses and crusts per section (0 = no crusts or abscesses; 1 = abscesses, no crusts; 2 = between 1 and 5 crusts, 3 = between 6 and 10 crusts, and 4 = more than 11 crusts). Neutrophil (MPO) abundance and T cell (CD3) abundance were determined by a semiquantitative analysis of dermal inflammation (0 = no inflammatory infiltrate; 1 = minimal inflammatory infiltrate; 2 = mild inflammatory infiltrate; 3 = moderate inflammatory infiltrate; and 4 = severe

inflammatory infiltrate). Data are displayed as the medians with interquartile ranges; whiskers indicate the min and max values (n = 4).

Figure S7

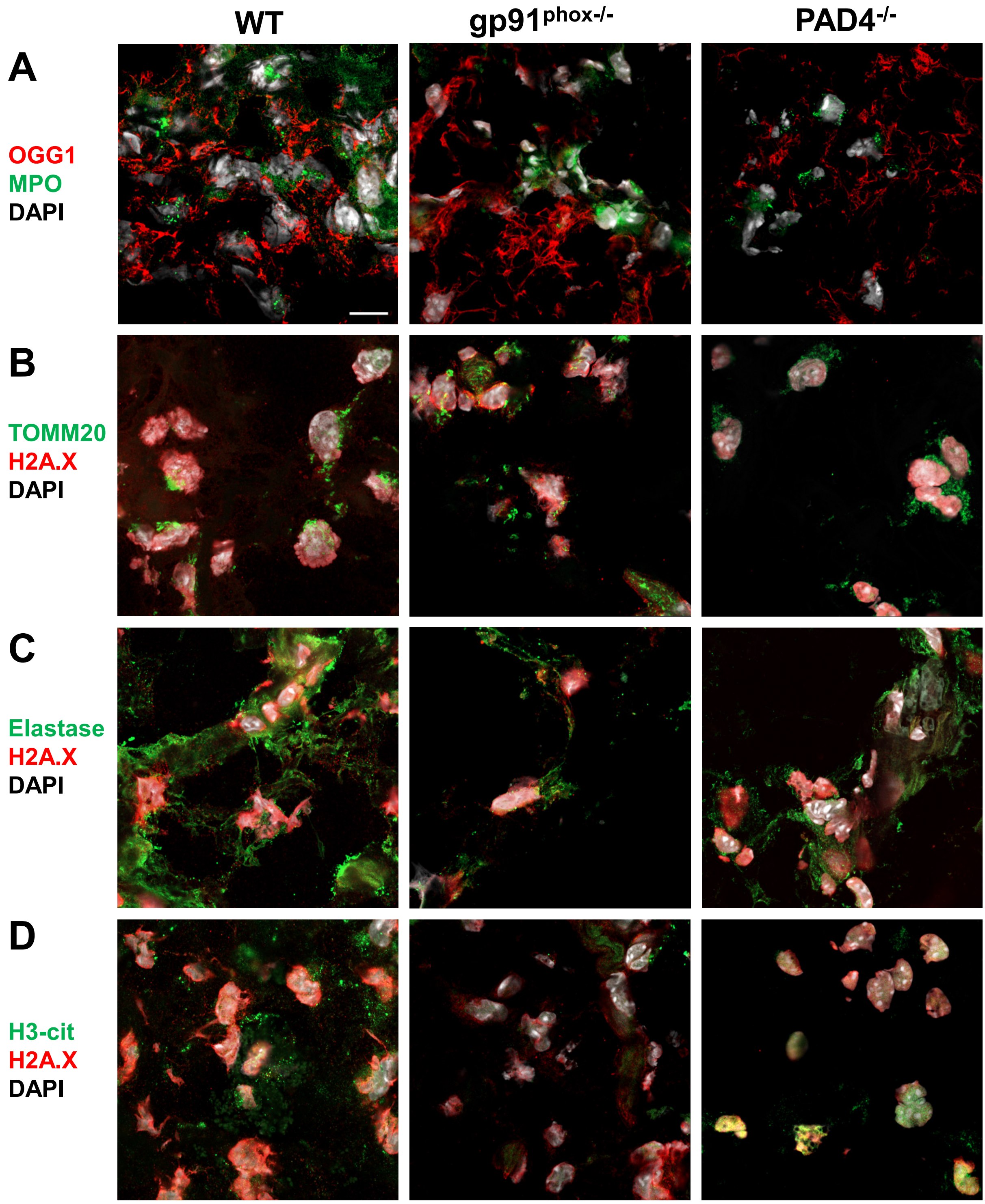

Immunofluorescence images of the ear tissue of WT, gp91$^{phox-/-}$ and PAD4$^{-/-}$ mice 7 h after the 1st challenge. **A**: red = OGG1; green = MPO; and gray = DAPI. **B**: red = HA2.X; green = TOMM20; and gray = DAPI. **C**: red = HA2.X; green = elastase; gray = DAPI. **D**: red = HA2.X; green = H3 citrullination; and gray = DAPI. For each experimental group and staining n = 3. The scale bar is 10 μm.

Figure S8

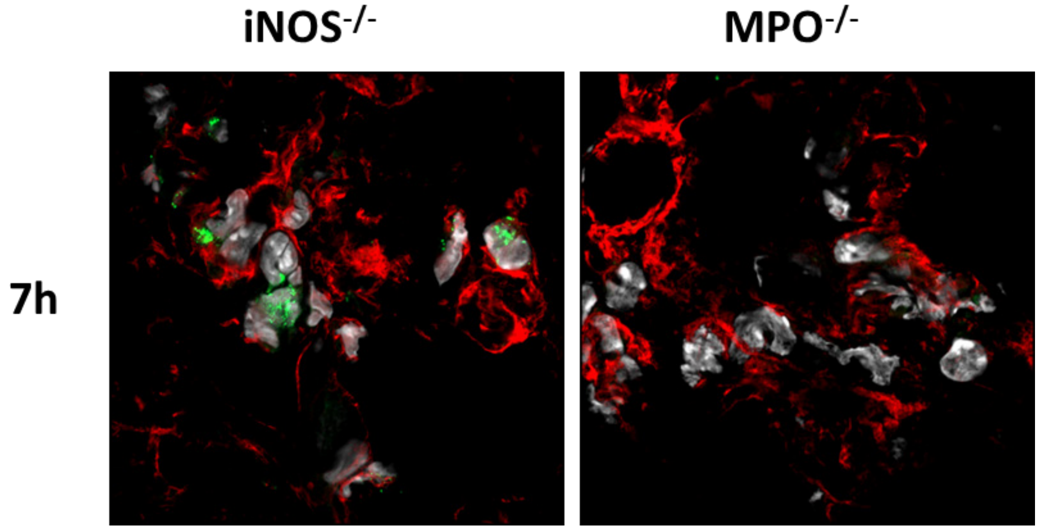

Immunofluorescence images of ear tissue of iNOS$^{-/-}$ and MPO$^{-/-}$ mice 7 h after the 1st challenge. Red = OGG1; green = MPO; and gray = DAPI (n = 3).
